# Dehalogenases: From Improved Performance to Potential Microbial Dehalogenation Applications

**DOI:** 10.3390/molecules23051100

**Published:** 2018-05-07

**Authors:** Thiau-Fu Ang, Jonathan Maiangwa, Abu Bakar Salleh, Yahaya M. Normi, Thean Chor Leow

**Affiliations:** 1 Department of Cell and Molecular Biology, Faculty of Biotechnology and Biomolecular Sciences, University Putra Malaysia, 43400 UPM Serdang, Selangor, Malaysia; ang.thiaufu@gmail.com (T.-F.A.); maiangwa@kasu.edu.ng (J.M.); normi_yahaya@upm.edu.my (Y.M.N.); 2 Enzyme and Microbial Technology Research Centre, Centre of Excellence, University Putra Malaysia, 43400 UPM Serdang, Selangor, Malaysia; abubakar@upm.edu.my; 3 Department of Biochemistry, Faculty of Biotechnology and Biomolecular Sciences, University Putra Malaysia, 43400 UPM Serdang, Selangor, Malaysia; 4 Institute of Bioscience, University Putra Malaysia, 43400 UPM Serdang, Selangor, Malaysia

**Keywords:** dehalogenases, applications, mechanisms, microbial

## Abstract

The variety of halogenated substances and their derivatives widely used as pesticides, herbicides and other industrial products is of great concern due to the hazardous nature of these compounds owing to their toxicity, and persistent environmental pollution. Therefore, from the viewpoint of environmental technology, the need for environmentally relevant enzymes involved in biodegradation of these pollutants has received a great boost. One result of this great deal of attention has been the identification of environmentally relevant bacteria that produce hydrolytic dehalogenases—key enzymes which are considered cost-effective and eco-friendly in the removal and detoxification of these pollutants. These group of enzymes catalyzing the cleavage of the carbon-halogen bond of organohalogen compounds have potential applications in the chemical industry and bioremediation. The dehalogenases make use of fundamentally different strategies with a common mechanism to cleave carbon-halogen bonds whereby, an active-site carboxylate group attacks the substrate C atom bound to the halogen atom to form an ester intermediate and a halide ion with subsequent hydrolysis of the intermediate. Structurally, these dehalogenases have been characterized and shown to use substitution mechanisms that proceed via a covalent aspartyl intermediate. More so, the widest dehalogenation spectrum of electron acceptors tested with bacterial strains which could dehalogenate recalcitrant organohalides has further proven the versatility of bacterial dehalogenators to be considered when determining the fate of halogenated organics at contaminated sites. In this review, the general features of most widely studied bacterial dehalogenases, their structural properties, basis of the degradation of organohalides and their derivatives and how they have been improved for various applications is discussed.

## 1. Introduction

Natural and man-made halogenated organic compounds have widespread applications in various industrial ventures as well as solvents in daily household items making them a significant class of environmental pollutants [1,2,3,4,5]. These halogenated compounds are of significant prominence in the marine ecosystem [6], and the extraordinary stability of the chemical bonds in these compounds makes their widespread occurrence mostly in the biosphere a persistent environmental concern. The halogenated compounds are the most utilized and studied groups, primarily due to the ease with which they are simply configured to facilitate biochemical processes [3]. The recalcitrant nature of these organic compounds and their toxicity even to microbes with the potential to degrade them makes their biodegradability practically difficult. Overexposure to most of these pollutants causes toxic and lethal accumulation leading to severe environmental and health consequences [7]. The decomposition and biodegradability of these compounds in nature are limited, and hence massive campaigns on remediation and recovery of polluted environments over the years are still ongoing.

Though organohalide-degrading microorganisms are difficult to find, microbial processes of rehabilitating halogenated polluted environments are the most implemented over the years [8]. The growing interest in the use of microbial processes in the removal and recovery of halogenated toxic polluted environments is practically due to their inducible enzyme system producing dehalogenases. Their remarkable survival abilities contribute to the importance of these microorganisms in pollutant degradation in complex and volatile halogenated environments. However, microorganisms have limited catabolic pathways for the complete mineralization of these compounds.

Microorganisms play important roles in remediating organohalide contaminated environments through naturally available or laterally evolved dehalogenases. These various dehalogenases catalyze the cofactor-independent dehalogenation under both aerobic and anaerobic conditions by the removal of the halogen substituent from toxic environmental pollutant [9,10,11]. Dehalogenases also have the potential to carry out other significant catalytic functions over a wide spectrum of substrates [12,13]. The dehalogenases have diverse organohalide substrate specificity including chlorinated, brominated and some iodinated substrate as well as the different length of substrates preference depending on the enzymes’ mechanism involved in degradation [14]. Their characteristic property and other physiological features determine the process conditions suitable for implementing transformation or mineralization processes by dehalogenation microorganisms [15]. Koudelakova et al. [14], summarized that general halogenated dehalogenases can effectively catalyze with catalytic activity in the range of 10^4^ to 10^5^ M^−1^s^−1^, while for certain anthropogenic multiple halogenated compounds like probable carcinogenic 1,2,3-trichloropropane [16], the catalytic activity is very low (40 M^−1^s^−1^). However, when a dehalogenation catalyzed reaction is initiated by an existing dehalogenase, persistence of toxic and/or highly reactive metabolic intermediates is imminent due to lack of enzymes for the rapid conversion of large amount these intermediate products [17].

Insight into the evolution, diversity and general mechanism of dehalogenase catalytic function obtained from biochemical and physical studies reveal an S_N_2 substitution mechanism involving a catalytic triad of Asp-His-Asp/Glu. Herein, carboxylate oxygen of the aspartate launches a nucleophilic attack on the carbon atom of the substrate bonded with halogen, producing a halide ion and alkyl-enzyme intermediate with an ester bond. The nearby His-Asp/Glu (acid-base pair) subsequently hydrolyzes a water molecule to produce a nucleophilic hydroxide that creates an attack on the carbon of the ester bond. This generates a tetrahedral intermediate which immediately decomposes to form RCH_2_O^−^, gaining a proton from the nucleophilic aspartate to form RCH_2_OH [14,18,19,20,21].

Structural studies of some dehalogenases have revealed and enabled a detailed understanding of a different intramolecular substitution mechanism and a hydratase-like mechanism, which also demonstrates a covalent aspartyl intermediate-mediated substitution mechanism [10,22,23]. All dehalogenases possess the unique feature of halide-binding residue(s) [24]. The halide-binding residue(s) is/are also known as halide-stabilizing residue(s), these residues are critical for the catalytic activity of dehalogenase as they help to stabilize the halide during formation of enzyme-substrate complex and important for leaving group stabilization [25,26]. Success in X-ray crystallographic analysis of some dehalogenases has provided impressive mechanistic and structural understanding of the mechanism of action, especially the intramolecular substitution mechanisms involved in the halogen atom removal [10,19]. As revealed in quantum mechanics and molecular simulations studies, the rate-determining step in the LinB-catalyzed degradation of 1,2-dichloropropane can be altered by water molecules in the reaction to influence the enantioselectivity of a *Sphingobium paucimobilis* UT26, LinB dehalogenase [27]. This review highlights some general features of most widely studied dehalogenases, their structural properties and how they have been improved for various applications.

### 1.1. Dehalogenases and Different Dehalogenation Processes

Dehalogenases enzymatic dehalogenation can be divided into several types such as reductive dehalogenation, oxygenolytic dehalogenation, dehydrohalogenation and hydrolytic dehalogenation. Dehalogenases share similar catalytic mechanisms involving a halogen replacement through nucleophilic substitution [28,29]. On these accounts, we shall briefly focus on occurrences, reaction mechanisms, and general features of reductive dehalogenation and hydrolytic dehalogenation commonly investigated in dehalogenation studies. Some of these dehalogenases are described in Table 1 below.

#### 1.1.1. Reductive Dehalogenation

Among the several dehalogenation, mechanisms are reductive dehalogenation, which occurs especially (but not exclusively) in anaerobic environments. Reductive dehalogenases (rdhA) are responsible for biological dehalogenation in organohalide respiring bacteria [30,31]. Details on remediation strategies resulting from microbial reductive dehalogenation have been recently reviewed [32]. The taxonomic and functional diversity of these key microorganisms and their reductive dehalogenase (rdhA) genes in some contaminated environments have also been reported [33,34]. The PCE-reductive dehalogenase (PCE-RDase from a dehalorespiring bacteria *Dehalospirillum multivorans* reduces PCE or TCE to *cis*-DCE as the terminal product [35,36]. A new pathway for the degradation of unsaturated aliphatic organohalogen compounds has been described for 2-halo-acrylate reductase [11]. Reductive dehalogenases form a distinct subfamily of cobalamin (B12)-dependent enzymes that are usually membrane associated and oxygen sensitive such as rdhAs from *Desulfitobacterium chlororespirans* and *Desulfitobacterium dehalogenans* [37,38]. Some membrane-associated chloroaromatic reductive dehalogenases have also been identified like 3-chlorobenzoate-reductive dehalogenase and 3-chloro-4-hydroxybenzoate dehalogenase [39,40,41,42]. Reductive dehalogenation, particularly in anaerobic environments of alkyl solvents to various extents, depends on the solvent, the physicochemical environment, and the microorganisms present [43]. Studies with rdhA from *Nitratireductor pacificus* pht-3B show that a direct interaction between the cobalamin cobalt and the substrate halogen underscores the catalytic mechanisms of the rdhAs [44]. Few enzymes acting on unsaturated aliphatic organohalogen compounds are described in dehalogenation of tetrachloroethene and trichloroethene by corrinoid/iron-sulfur-cluster-containing reductive dehalogenases [45], and the hydration of *cis*- and *trans*-3-chloroacrylate, respectively, by cofactor-independent *trans*- and *cis*-3-chloroacrylic acid dehalogenase (CaaD and *cis*-CaaD, respectively) belonging to the β-α-β fold of the tautomerase superfamily [10,46].

#### 1.1.2. Hydrolytic Dehalogenation

**Figure 1:**
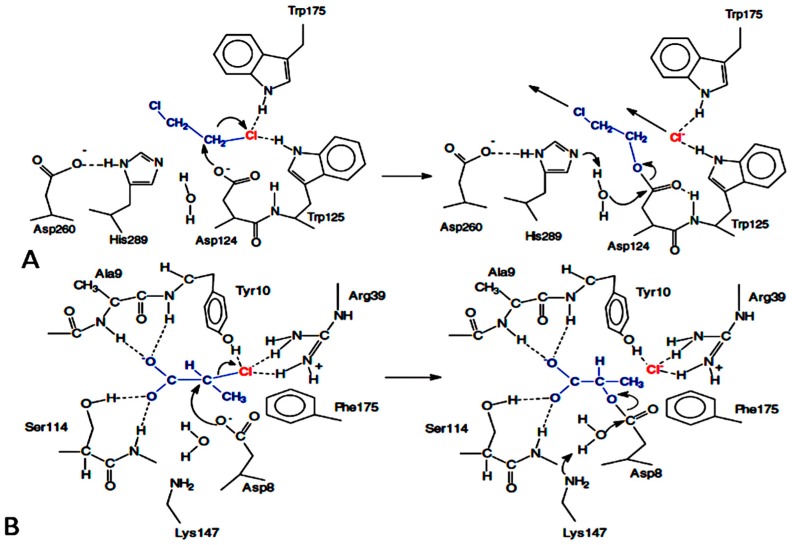
Simplified scheme of catalytic mechanism for aliphatic organochlorine compounds (**A**) haloalkane dehalogenase (HLD) and (**B**) HAD-type haloacid dehalogenase (DhlB) [11].

Hydrolytic dehalogenation is commonly performed by haloalkane dehalogenase, 2-haloacid dehalogenase, 4-chlorobenzoyl-CoA dehalogenase and fluoroacetate dehalogenase [10,11]. Bromoacetate dehalogenase has also been described [47]. The well-studied group of hydrolytic dehalogenases are the haloalkane dehalogenases and 2-haloacid dehalogenases and their reaction mechanisms are shown in Figure 1 [15,48]. These dehalogenases required water to break down the ester intermediate formed after nucleophile aspartate attacks the alpha carbon of halogenated compounds. Various metabolic pathways for saturated organohalogen compounds and aromatic organohalogen compounds have been described for several bacterial hydrolytic dehalogenases and related enzymes, particularly the haloalkane dehalogenase(HLDs), haloacid dehalogenases (HAD), fluoroacetate dehalogenases (FAD), 4-chlorobenzoate-coenzyme A dehalogenases (COA), and haloacrylate reductase [11,49], which are briefly described.

#### 1.1.3. The Haloacid Dehalogenase

Haloacid dehalogenase (HAD)-like enzymes comprise a large superfamily of phosphohydrolases present in all organisms. The 2-haloacid dehalogenases are further classified into three types based on their substrate specificities. The l-2-haloacid dehalogenases (l-DEX) catalyze the dehalogenation of l-2-haloalkanoic acids to produce the corresponding d-2-hydroxyalkanoic acid. The d-2-haloacid dehalogenases (d-DEX) act on d-2-haloalkanoic acids to yield l-2-hydroxyalkanoic acids, and the d,l-2-haloacid dehalogenases (d,l-DEX) react with both d- and l-2-hydroxy-haloalkanoic acids, producing l- and d-2-hydroxyalkanoic acids, respectively [50]. There are two groups of unique haloacid dehalogenases which have been better characterized as Group I and II α-Haloacid dehalogenase based on their different substrate intermediate catalytic mechanisms. While hydrolytic water performs a direct nucleophilic attack on the substrate α-carbon, displacing the bound halogen in Group I, the reaction proceeds through an esterified intermediate hydrolysis in Group II [23,51,52,53,54,55,56,57,58]. Biochemically characterized yeast phosphatases from the haloacid dehalogenase superfamily active against various phosphorylated metabolites and peptides and implicated in detoxification of phosphorylated compounds and pseudouridine have been reviewed [59]. Haloacid dehalogenases activity is said to exist in other enzymes, including oxygenases, dehydrogenases, and hydrolases which have also been shown to act on the degradation of polychlorinated biphenyls [60].

#### 1.1.4. The Haloalkane Dehalogenase

**Figure 2:**
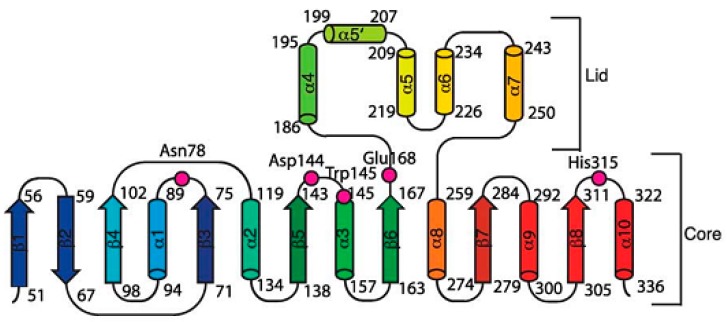
Topology diagram of haloalkane dehalogenase. This is an α/β-hydrolase fold structure. The residues labelled are involved in catalysis. Note that all of the catalytic residues are located at the linker [21].

Haloalkane dehalogenases convert haloalkanes to their corresponding alcohols and halides and protons [14,61,62]. They can break the carbon-halogen bond in halohydrocarbons through a hydrolytic dechlorination mechanism [63]. The HLDs belong to a family of α/β-hydrolase fold which contains seven parallel and one anti-parallel β-pleated sheets which are flanked by α-helices (Figure 2), which is also known as the core domain where the dehalogenation activity takes place. The structural features bores slight similarity to the l-2-haloacid dehalogenase [53]. The main domain is followed by continuous 5 α-helices of the lid domain, which form a cap structure and in between is the active site. The core domain also plays significant role in structural stability [64]. The active site is mainly flanked by hydrophobic residue cavity buried between the main domain and the cap domain of the enzyme [18,21,65]. The dehalogenation process catalyzed by haloalkane dehalogenase can be executed without oxygen or any other cofactor except water, which makes it of interest for several biotechnological purposes [66,67]. However, deprotonation of His^272^ increases binding of anions in the access tunnel, and the anionic ordering does not change with the switch of the protonation state on the process of halide release [68]. HLDs catalysis basically follows the S_N_2 substitution mechanism involving a catalytic Asp-His-Asp/Glu triad and two halide-stabilizing residues (Trp-Trp or Trp-Asn). A major deviation in the catalytic groups of HLDs and other non-hydrolytic enzymes such as the 4-chlorobenzoyl-coenzyme A dehalogenase is seen with the essential tryptophan residues in which two Trp^175,125^ residues form a binding pocket for the incipient halide ion [19]. Initially, carboxylate oxygen of the aspartate launches a nucleophilic attack on the carbon atom of the substrate that is bonded with halogen [14,18,19,20,21,61,69,70]. It can be clearly seen from that the aspartate acts as nucleophile and histidine as base for different type of haloalkane dehalogenases but the acid can be glutamate or aspartate and the two halide-stabilizing residues can be any combination formed by tryptophan and asparagine mostly. The positioning of Leu^177^ at the entrance tunnel to the active site makes the haloalkane dehalogenase-like proteins pocket residues most variable among the dehalogenases [71].

#### 1.1.5. The Fluoroacetate Dehalogenase

**Figure 3:**
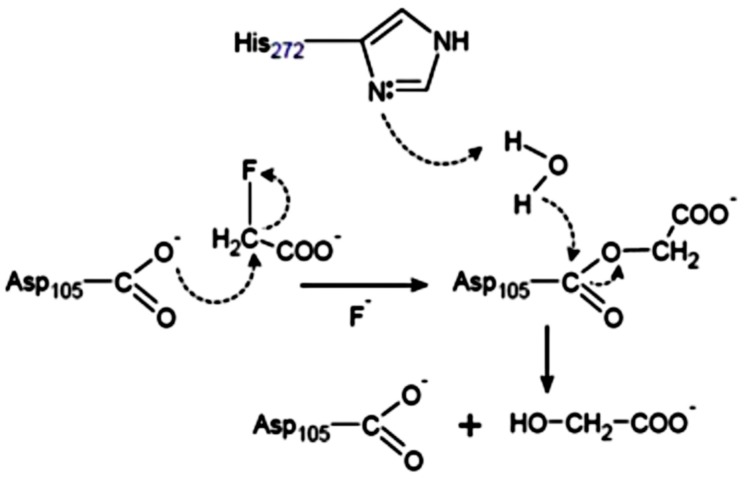
The dehalogenation by a fluoroacetate dehalogenase from *Delftia acidovorans* [80].

Fluoroacetate dehalogenases (FAcDs) catalyze the dehalogenation of fluoroacetate and some also cleave chlorinated and brominated analogues at slower rates. Considered the strongest covalent bond, the carbon-fluorine bond is readily hydrolyzed by fluoroacetate dehalogenases. This process requires a halide pocket which supplies hydrogen bonds to stabilize the fluoride ion, and also orchestrated to fine-tune the smaller fluorine halogen atom to establish selectivity towards fluorinated substrates [72]. Hydrolysis of various short-chain 2-haloacids with fluoroacetate (FAc) has been substantiated in the first fluoroacetate dehalogenase from a pseudomonad [73,74,75]. Ejection of a fluoride ion is carried out by an aspartate nucleophile in an S_N_2 of FAcDs defluorination activity. The FAcDs are structurally homologous to the non-defluorinating, haloalkane dehalogenases as well as the l-2-haloacid dehalogenases [10,11,19,54,76]. The Asp^105^ from fluoroacetate dehalogenase from *Moraxella* Sp. B accounts for S_N_2 mechanism involving an ester intermediate and together with nearby His^272^ with nearby tryptophan, they are proposed to have stabilizing function. And by way of supporting further fluoride stabilization, additional tyrosine residue takes part in the cleavage of the C-F bond predictively [77]. The important role of residues His^109^, Asp^134^, Lys^181^, and His^280^ have also been predicted to be useful in de novo enzyme designing in enhancing the C-F or C-Cl bond cleavage [78]. Several soil bacteria play a role in defluorination of fluoroacetate, and the fluoroacetate dehalogenase enzymes identified in some of these bacteria appear to degrade fluoroacetate via a similar mechanism (Figure 3), and more strikingly, bacteria harboring two haloacetate dehalogenase enzymes—fluoroacetate dehalogenase H-1 (dehH1) and fluoroacetate dehalogenase H-2 (dehH2)—have only been described in *Delftia acidovorans* strain B [79,80]. The amino acid sequence of fluoroacetate dehalogenase from *Moraxella* Sp. B is similar to that of haloalkane dehalogenase from *Xanthobacter autotrophicus* GJ10 [81]. Moreover, Asp^124^ and His^289^ of the haloalkane dehalogenase from *Xanthobacter autotrophicus* GJ10 serving as the nucleophile and the base, respectively, which correspond to Asp^105^ and His^272^, respectively in the fluoroacetate dehalogenase from *Moraxella* Sp. B [82].

#### 1.1.6. The 4-Chlorobenzoyl CoA Dehalogenase

**Figure 4:**
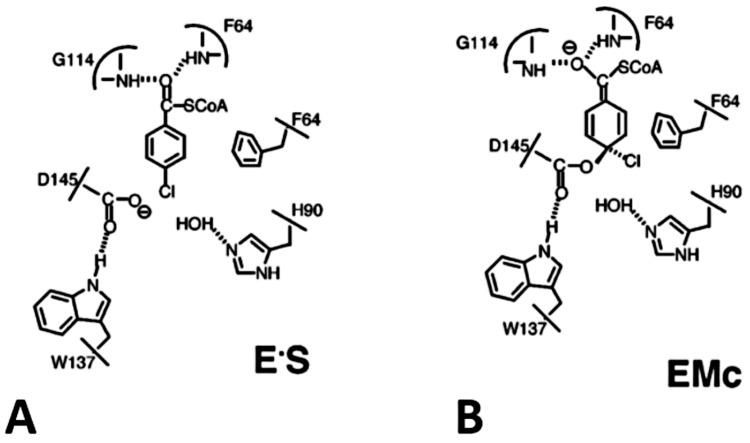
Steps of 4-chlorobenzoyl-CoA dehalogenase catalysis. The catalytic residues functioning in the enzyme-substrate (**A**), the Meisenheimer intermediate (**B**) are shown.

The 4-chlorobenzoyl-CoA dehalogenase whose catalytic function is dependent on coenzyme A and ATP, catalyzes the hydrolytic dehalogenation of 4-chlorobenzoate-CoA to 4-hydroxybenzoyl-CoA followed by abstraction of the chloride ion to form an arylated enzyme intermediate (EAr) and then ester hydrolysis. As opposed to non-enzymatic systems where the general-base catalysis by acetate ions facilitates attack by water, the initial attack in 4-chlorobenzoyl CoA dehalogenase catalyzed reactions is facilitated by the carboxylate of nucleophilic Asp^145^ on C (4) of the substrate benzoyl ring which gives rise to the Meisenheimer intermediate (EMc) (Figure 4) [83,84]. 

A two-fold advantage is derived from these phenomena, enhancement of the strength of the nucleophile and decreasing the entropy of activation [9]. The 4-chlorobenzoyl CoA dehalogenase also utilizes an active-site carboxylate to displace chloride from 4-chlorobenzoyl CoA which most likely proceed by the S_N_Ar mechanism for the dehalogenation reaction [85]. Although it’s molecular architecture bears no resemblance to that of other HLDs and l-2-haloacid dehalogenases, dehalogenation in the 4-chlorobenzoyl CoA dehalogenase proceeds in a two-step mechanism, with an aspartate residue as the active nucleophile in the first step of the reaction [19,53]. The dynamics of the active site indicates that water molecules entry into the active site tunnels forms a hydrogen bond with Asp^14^. Aromatic residues with favorable orientation provides a stability cradle for binding interactions with halide ion in 4-chlorobenzoyl-CoA dehalogenase [19,86]. The catalytic effectiveness of 4-chlorobenzoyl CoA dehalogenase catalyzed dehalogenation reaction is unique, considering the notoriety of aromatic substitution reactions on unactivated aromatic rings.

### 1.2. Microbial Dehalogenation and Their Significant Properties

Anaerobic, aerobic, and hydrolytic anaerobic microbial dehalogenation can take place as a respiratory process, referred to as organohalide respiration (OHR) where the halogenated substrate is used as terminal electron acceptor, but also as a general step in catabolic processes. Microorganisms noted for their remarkable ability to degrade, partially or fully, singly and/or in consortia, these halogenated compounds have been described to produce hydrolytic dehalogenases with a wide spectrum of degradative efficiency (Table 1). The utilization of these organic pollutants by microorganisms follows several phases of extracellular emulsification, periplasmic dehalogenation, and intracellular degradation of the residual carbon skeleton [3]. Interestingly, other forms of organisms such as the *Drosophila* Sp. have been demonstrated to produce alcohol dehalogenases [87]. It is significantly shown that certain genome rearrangements characteristic is connected with the expression of variety of dehalogenase gene, which contributes to their acquisition of biodegradation pathways for halogenated compounds as described in a haloalkane-utilizing bacterium *Rhodococcus rhodochrous* [88].

The effectiveness of the vast majority of the organohalide-utilizing microbes causes severe metabolic stress arising from the toxicity of organohalide compounds. As described for a haloalkane dehalogenase (DhlA) producing Gram-negative *Pseudomonas pavonaceae*, the high toxicity resulting from the aerobic catabolism of 1,3-dichloroprop-1-ene (1,3-DCP; a carcinogenic organohalide) causes a physiological restriction of the bacterium in the appropriate balance of NADPH/NADPþ, which is a central feature to the continued performance of virtually any aerobic microorganism biodegradation of organic halides. An appropriate supply of NADPH ensures rapid growth and provides the reducing power for the microbes to halogenate organohalides as their carbon source [17]. This in the past was demonstrated in an induced NADH stimulated oxygen dependent cell-free dehalogenation by *Pseudomonas* Sp. strain 273, producing dichloroalkane-dehalogenase [8]. Physiological restraints by other halogenated compounds have caused effective halogenating activity failure to cell of *Athrobacter* Sp. with three inducible haloalkane dehalogenases [89]. However, the transient accumulation of phenol as an early intermediate in the degradation of a hydrophobic recalcitrant halogenated bromobenzene (BrB) by the extracellular dehydrogenase from a tropical marine yeast *Yarrowia lipolytica* 3589, is said to proceed without any physiological restriction on the yeast growth. The yeast cells could grow aerobically on the degradative intermediate compounds through hydrophobic and acid-base interactions with a corresponding increase in cell mass [6].

The versatility of *Arthrobacter* Sp. in the degradation of the vast majority of environmental pollutants has been described to be ultimately due to their ubiquity in extreme environments and ability to produce halogenating metabolites [90,91]. They can tolerate long-term starvation and other metabolically challenging factors due to their ability to produce alcohol-dehalogenase in the degradation of excess heavy metals and toxic chemicals such as nitroglycerin, benzene derivatives, polycyclic aromatic compounds, haloalcohols, haloalkanes, *N*-heterocyclic compounds, insecticides, and herbicides [92,93,94].

In whole-cell biocatalysis, genetically transcribed dehalogenases such as diguanylatecyclase (yedQ) or c-di-GMP phosphodiesterase (yhjH) of *Pseudomonas putida* to form biofilms can be programmed for higher biochemical dehalogenase activity towards chlorobutane degradation in aerobic conditions [128]. In the earlier days, methane-utilizing bacteria have been used to halogenate chlorobutane as a sole carbon and energy source to release halogens under anaerobic conditions. However, with *Pseudomonas butanovora*, halogenation could be achieved aerobically with all reactions mediated by the oxygenase and halidohydrohlase dehalogenases [129]. Clinical human parasites of the *Mycobacteria* Sp. such as *Mycobacterium tuberculosis* H37Rv has demonstrable dehalogenation potency towards chlorinated and brominated haloaliphatics, as mediated by their hydrolytic dehalogenase [130].

The plasmid coding genes for dehalogenases (DhaA_f_ and DhaA) have been described for some bacteria, which reveals a shared relationship in regions fused to a fragment of a haloalcohol dehalogenase among strains able to degrade chlorinated and brominated haloalcohols in *Mycobacteria*, *Rhodococcus rhodochrous* and *Corynebacterium* spp. [131,132]. Recently, a functional dehalogenase (DadB) from *Alcanivorax dieselolei* B-5 DadB was revealed to possess a large access tunnel which allows entry of large halogenated compounds to the active site cavity, hence its wide spectrum of substrate specificity [112]. The *Sphingobium paucimobilis* UT26, LinB dehalogenase has a wide substrate specificity as revealed by its correlated binding affinity with hydrophobicity, as well as its molecular surface and dipole [133]. Particularly, the ability of LinB to catalyze the process of dehalogenation in the absence of any cofactor except water in addition to its nonlinearity with substrate size and variability of its binding affinities makes it of significant interest in dehalogenation processes [134,135,136]. Haloacrylate hydratase (Caa67_YL_) from *Pseudomonas* Sp., a new class of dehalogenase belonging to the flavour enzyme family can mediate the halogenation of organohalogen anaerobically in the presence of FAD and a reducing agent [NAD(P)H or sodium dithionite] and water [137].

Studies on a homologous protein designated (Cg10062) from *Corynebacterium glutamicum* has provided insight into the evolutionary emergence of the *cis*-CaaD and its catalytic proficiency as a dehalogenating enzyme in the tautomerase superfamily [46,138]. The bacteria of the *Burkholderia* Sp. and *Pseudomonas* Sp. reported utilizing 2-chloroacrylate as their sole carbon source have been described to inducibly synthesize 2-haloacid dehalogenases enzymes catalyzing the conversion of 2-chloroacrylate into 2-chloropropionate in the presence of NADPH [103,139,140]. Cryptic haloalkanoic acid dehalogenase genes from *Agrobacterium tumefaciens* (DhlS5II and DhlS5I) and *Burkholderia cepacia* (Chd1) makes use of a variety of distinctly different catalytic mechanisms in catalyzing halide hydrolysis with inversion of product configuration [101,141].

Another group of dehalogenases derived from *Pseudomonas* Sp. and *Methylobacterium* Sp. is the haloacid dehalogenase. Prominently acting on the chiral carbon atoms of the d- and l-2-haloakanoic acid enantiomers make the d,l-2-haloacid dehalogenase significant in the chemical conversion of both enantiomers of the substrates as compared to the racemases enzymes [50,142,143]. Generally, the uniqueness of the d,l-2-haloacid dehalogenase to other hydrolytic dehalogenases is that the overall reaction rates are controlled by different catalytic steps without the formation of an ester intermediate [11,15,50]. An important example of stereoconfiguration in dehalogenases is described in the *Rhizobium* Sp. RC1 d-haloalkanoic-specific dehalogenase (DehD) and HadD from *Pseudomonas putida* AJ1 whereby they catalyze the hydrolytic dehalogenation of d-haloalkanoic acids with inversion around the chiral carbon [144]. The α-haloacid dehalogenases have also been described for *Pseudomonas putida* strain PP3 with two different dehalogenating mechanisms described for its dehalogenases (DehI and DehII) [145]. These dehalogenases catalyze substitution reactions at their chiral centres leading to the removal of halides from d-haloalkanoic acids and in some cases also the l-enantiomers [99].

Discovered in some bacteria of *Pseudomonas* strains, *Delftia acidovorans* strain B (formerly *Moraxella* Sp. strain B), *Streptomyces cattleya*, and *Burkholderia* Sp. FA1 [74,79,81], haloacetate dehalogenases genetic modification and recombinant expression of this enzyme have significantly alleviated the toxicological problem of fluoroacetate poisoning in animals. The transformation of *Saccharomyces cerevisiae* has been demonstrated with the application of gene coding for fluoroacetate dehalogenase as a selection pressure marker [146,147,148]. Novel hydrolytic 4-chlorobenzoyl–coenzyme A (CoA) dehalogenase from (chlorothalonil)-degrading strain of *Pseudomonas* Sp. is reported as the only dehalogenase that catalyzes halogenation of aromatics via a coenzyme precursor. However, the Chlorothalonil hydrolytic dehalogenase (Chd) from the same *Pseudomonas* Sp. is reported to catalyze halogenation of aromatics independent of coenzyme A and ATP [9,149].

The anaerobic bacterium *Dehalococcoides ethenogenes* is the only known organism that can completely dechlorinate tetrachloroethene or trichloroethene (TCE) to ethene via dehalorespiration. The persistence of these halogenated compounds for ancient centuries in the environment is proposed to have driven the evolution of dehalorespiration and the associated dehalogenases in them [122]. The organohalide-respiring *Dehalococcoides* (Dhc) Sp. have significant remediating power by anaerobically catalyzing organohalide respiration through the enzymatic reductive dehalogenases [122,123,150]. Other organohalides respiring strains of *Dehalobacter*, *Sulfurospirillum*, *Desulfitobacterium* and *Dehalogenimonas* Sp. have been described [151,152,153]. Transcription analysis of the rdhA genes (reductase gene transcripts), unravelled several reductive dehalogenases genes with diverse organohalide substrate specificity. About six of the rdhA genes coding for several dehalogenases have been demonstrated to be up-regulated and expressed for functional remediation potential [154]. Reductive dehalogenases have been genetically and biochemically described to adopt an extra-cytoplasmic corrinoid-containing iron-sulphur proteins [155]. Genetic and biochemical studies have provided insights into dehydrohalogenation of polyhalogenated compounds mediated by dioxygenase during which the cleavage of catechol (carbon-chlorine bonds) precedes dehalogenation as described in dioxygenase TecA of *Burkholderia* PS12 [156]. In other metabolic dehalogenations, reductive displacement of a chlorine from a ring structure by a glutathione-dependent reaction is also described for a tetrachlorohydroquinone reductive dehalogenase (TCD) in *Sphingomonas chlorophenolica*, and it is proposed that the eukaryotic maleylacetoacetate isomerase is homologous to the reductive dehalogenase in *S. chlorophenolica* and *S. paucimobilis* [157]. The extraordinary versatility of TCHQ dehalogenase can be attributed in part to the active site architecture typical of the GST superfamily, in which most of the residues that contribute to glutathione binding and ionization are provided by the *N*-terminal domain, while most of the residues that contribute to substrate specificity are provided by the *C*-terminal domain [157,158].

Other sources of halogenases have also been described from the bromophenol producing marine sponge *Aplysina aerophoba* [159]. Recent studies on the genomes and genetic information on the diversity of genes encoding for unspecific halogenases as well as metabolic and cometabolic dehalogenases in the soil metagenome have shown large genera of microbial contribution to dehalogenation processes particularly in the *Bradyrhizobium* Sp. [160]. Group of other halogenating enzymes also catalyze halogenation reactions such as the haloperoxidases using hydrogen peroxide and a halogen ion as substrate [161]. Microorganisms inhabiting the terrestrial environments such as fungi have the potential to halogenate organic compounds. This is evidenced in the first discovered heme-chloroperoxidase (CPO) from the terrestrial fungus *Caldariomyces fumago* and other novel fungal families [162,163]. Besides their ubiquitous phenomenon of halogenating volatile organohalogens, the methyltransferase of fungi is said to contain halomethanes [164].

### 1.3. Structural Studies on Dehalogenases and Reaction Mechanisms

The earliest structural studies of dehalogenases demonstrated that the haloalkane and the haloacid dehalogenases are hydrolytic enzymes with evolutionary relationship to other α/β hydrolase enzymes [22,23,165]. Similarly, crystallographic studies have shown that dehalogenase catalysis mechanistically proceeds in a two-step ester covalent bond formation with an active site nucleophile aspartate and base histidine (Table 2) [19]. The two-step route for halide release involves transfer from the halide-binding site in the cavity to a binding site at the protein surface forming a collision complex and conformational changes resulting in a more open configuration of the active site allowing a readily escape of the halide ion (Table 2) [26]. These steps have been detailed on three different substrates using a combination of quantum mechanical calculations and molecular dynamics simulations [166].

The active site orientation and protonation states likely to be involved in catalysis can be properly understood from structural and simulation studies on dehalogenases. Structural sequence annotation has also revealed a wide difference in conserved residues similarity between the dehalogenases and dehydrogenase/reductase (SDR) family [87]. Aliphatic dehalogenases formed by the halohydrin dehalogenases has been structurally investigated to be somewhat similar to the members of the short-chain dehydrogenase reductase (SDR) superfamily of proteins in their reaction mechanisms [167]. The complexity and uncertainties in their structure-function relationship are of importance in distinguishing how the arrangements of secondary structural elements do not profit similar functions [168]. Despite these low similarity, the dehalogenases still share some structural and mechanistical relationship to the short-chain dehydrogenases/reductases in their conserved catalytic triad Ser, Tyr and Lys/Arg residues [167]. The structural details obtained in the absence of the active site Tyrosine (Tyr) suggests that the short-chain dehydrogenase/reductase (SDR) family could also be involved in other functions besides enzymic reactions [169].

Most haloacid dehalogenases share a structurally distinct α/β hydrolase fold from that of other hydrolase family enzymes. The helical domains observed by x-ray structures of the mesophilic L-2-haloacid dehalogenases (l-DEX YL) from *Pseudomonas* Sp. YL and DhlB from *Xanthobacter autotrophicus* GJ10 provide a tight homodimeric interface which limits substrate specificity. Because of this these dehalogenases have a much more enclosed active site and have been shown to only accept small substrates up to the size of chloropropionic acid [23,53,97]. While dimerization may facilitate structural stability in these enzymes, the disulphide bond is said to facilitate the structural stabilization of the monomeric form in haloacid dehalogenase PH0459 from *Pyrococcus horikoshii* OT3 [52]. The l-2-haloacid dehalogenase from the thermophilic archaeon *Sulfolobus tokodaii* adopts an oligomeric state by structure resolution and lacks cysteine residues, hence incapable of using this mechanism of stabilization [107]. In the structural studies of the haloalcohol dehalogenase HheC from *Agrobacterium radiobacter* AD1, the reactions catalyzed when complexed with a bound haloalcohol substrate mimic, reveals formation of an intramolecular nucleophile that substitutes the vicinal halogen when the haloalcohol hydroxyl is deprotonated by a substitutive process with a halide-binding site at the location of the NAD(P)H binding site [10,170,171,172]. In the structural determination of l-2-haloacid dehalogenase from *Xanthobacter autotrophicus* GJ10 complexed with l-2-mono-chloropropionate and monochloroacetate, the nucleophilic Asp^8^ interaction with surrounding charged and polar amino acids constitutes the formation of a covalent enzyme-ester intermediate which is subsequently hydrolyzed by water [173]. Similarly, the residues spanning the active centre accounts for the limited substrates specificity and stereospecificity [23,97]. These residues are proposed to be ultimately involved in the binding of the substrate carboxyl moiety as well as the formation of a halide-binding cradle based on the active centre position of the substrate formate ion [53].

The 4-chlorobenzoyl-coenzyme A dehalogenase has a specialized mode of catalysis in which an active site carboxylate side chain is employed in the displacement of the chloride ion resulting in the formation of an arylated enzyme intermediate [85]. The polarization of the thioester carbonyl activates the benzoyl ring C(4) toward nucleophilic attack by Asp^145^ and stabilizes the resulting Meisenheimer intermediate [84,166,174]. X-ray structural studies on the metal-dependent halogenase enzymes chlorohydrolase AtzA and TrzN showed they exist in their hexameric and dimeric form, respectively, and the active-site metal Fe^2+^ atom though being the physiologically relevant cation in AtzA is bound but not tightly coordinated in the active site and yet less tightly coordinated than Zn^2+^ cations in TrzN. The observed mutations conferred on the enzyme by natural selection (Ala170Thr, Met256Ile, Pro258Thr and Tyr261Ser) located in the interface is responsible for coordinating the hexameric form of the enzyme [175]. Furthermore, accessibility of substrate-binding pocket and catalytic centre is through a hydrophobic channel, and the orientation of the binding pocket in the AtzA will most likely not permit nucleophilic substitution by an activated water, whereas, Thr^325^ is uniquely positioned instead of a conserved aspartate that ligates the Zn^2+^ cations in TrzN [176]. The enzymes also have contrasting metal-dependent reactions mechanisms with other known dehalogenases wherein they use an active-site carboxylic acid (Asp) to displace the halide ion [19,175].

Although the *Xanthobacter* and *Rhodococcus* haloalkane dehalogenases share 30% amino acid sequence identity, the structural resolution has shown significant structural homology in the α/β-hydrolase core, with the difference in their catalytic triad positioning, and the substrate and product-binding site [65]. In the conformational analysis of haloalkane dehalogenase LinB from *Sphingomonas paucimobilis* UT26, the charge state and double-protonation of the catalytic base (histidine), could be a determinant in the geometry distortion of the catalytic nucleophile (aspartate) [12]. The structural complexes of LinB with 1,2-propanediol/1-bromopropane-2-ol and 2-bromo-2-propene-1-ol, debromination of 1,2-dibromopropane and 2,3-dibromopropene, respectively, conformed to the observed general trend that the sp^3^-hybridized carbon is the predominant electrophilic site for the S_N_2 bimolecular nucleophilic substitution in dehalogenation reaction [177]. The S_N_2 imposes a collinear alignment of the nucleophile and electrophile to any displaced halide further impacting the stabilization role of the dehalogenase catalytic triad [72]. With majority of its disordered residues found on the surface, the core domain of LinB appears to be very stable with little anisotropy in residue motions, and flexible regions marked with higher B factors leading to the active site, allow entry of large substrates into the active site with isotropic B-factors of buried atoms ranging from 2.5 Å to 8.1 Å. These residues also show stabilization of the halide is weaker in LinB compared with DhlA, and the affinity of LinB for halide is also not high [12]. The major determinant of the substrate specificity of this haloalkane dehalogenase based on the structural features involves arrangement and composition of the *R*-helices in the cap domain in adaptation toward xenobiotic substrates [61].

The mechanism in carbon-halogen bond cleavage is mediated by a hydrated catalytic activation of the active site residues. Although structurally, the catalytically active residues differ among the fluoroacetate dehalogenase and other dehalogenases (Table 2), halide pocket requirement is crucial for the supply of hydrogen bonds and the active site dynamics of the enzyme. Structural studies of the fluoroacetate dehalogenase FAcD has revealed how the hydrogen bonds of the halide pocket stabilize the fluoride ion, which is also tailored for selectivity towards enzymatic defluorination of fluorinated substrates [72]. The mechanism for these hydration has been described in the structural elucidation of how the altered active site environment of trans-3-chloroacrylic acid dehalogenase (CaaD) from *Pseudomonas pavonaceae*-170, facilitates the hydration of the α,β-unsaturated bonds of *trans*-3-chloroacrylate and 3-bromopropiolate [102] This reaction involves the hydrolytic cleavage of the carbon-halogen bond to the corresponding product, whereas for other unsaturated organohalogen compounds a co-factor dependent reduction of the carbon-carbon double bond is required [139]. In the native *cis*-CaaD and inactivated *cis*-CaaD structural elucidation, comparison of the two structures highlights their different substrate specificities in which substrate binding is supported by an additional carboxylate residue (His^28^) in *cis*-CaaD and with covalent modification of the Pro^1^ nitrogen atom in the inactivated *cis*-CaaD. The orientation of the substrate towards the active site of these two structures distinguishes their structural basis for substrate specificity and inactivation by (*R*)-oxirane-2-carboxylate [178].

Structure resolution of dehalogenase carried in complex with corresponding chain length substrates has enabled a better understanding of the reaction mechanisms for most dehalogenases. The d,l-2-haloacid dehalogenase and d,l-DEX convert both enantiomers of organohalogenated substrate. However, the chlorine isotope effects of this enzyme have been described structurally to precede the dehalogenation step [179]. This is more so, that most dehalogenases share different active site residue positions. In a structural resolution of a d,l-2-haloacid dehalogenase complexed with the substrate-analogue, catalytically relevant residues appear shielded away from the substrate hence explaining its limited substrate specificity [53]. When substrates are complexed with enzymes, the overall polypeptide fold of the enzyme is structurally maintained such that orientation of catalytically important residues around the alkyl groups of substrate moieties serves to enhance stabilization through hydrophobic interactions, and determines the stereospecificity of the enzyme. Importantly, the trapped water molecule in the vicinities of the carboxyl carbon of the active site pocket residues may hydrolyze the ester intermediate and its substrate [104]. This phenomenon has been described in the structural studies of DelVa dehalogenase from *Burkholderia cepacia* MBA4 where the positional orientation of the water molecule influenced ester hydrolysis [54].

Structural details of the group II haloacid dehalogenase have been well described, however not until recently only the functionally important residues of a representative group I αHA dehalogenase have been identified in d,l-DEX 113 [51]. The DehI processes both l- and d-substrates and three possible sites for halide binding have been predicted within the DehI active site. Although there are no structural homologues of the DehI in the structural databases, the first reported structure of a group I αHA dehalogenase have revealed an electrostatic surface around the binding cavity that is highly basic, relative to an exclusively acidic molecular surface [54]. This property attracts negatively charged halo-acids to the entrance of the active site. It was experimentally proposed that the DehI α-HA dehalogenase enzymes do not utilize a reaction mechanism analogous to that of the group II αHA dehalogenases or the haloalkane dehalogenase [177]. Preliminary X-ray crystallographic analysis of DehI, a group I α-haloacid dehalogenase from *Pseudomonas putida* strain PP3 [99] proposes an alternative mechanism in which the activation of a water molecule for nucleophilic attack of the substrate chiral center resulting in an inversion of configuration of either l- or d-substrates contrasts with Group II enzymes [51,99]. The nucleophilic attack of the substrate occurs via an activated adjacent water molecule which is activated by either Asp^189^ or Asn^114^ with the formation of an S_N_2 transition-state intermediate followed by the release of the halide and the formation of the hydroxylated product inverted about the alpha carbon (Cα). Reports have described the l-2-haloacid dehalogenase from the thermophilic archaeon *Sulfolobus tokodaii* homodimer with each monomer being composed of a core-domain of a β-sheet bundle surrounded by α-helices and an α-helical subdomain. The salt bridge between Asp^7^ and Lys^128^ increases the nucleophilicity of the catalytic aspartate [107]. Similarly, in the structural studies of a group I putative l-haloacid dehalogenase gene (DehRhb) from a marine *Rhodobacteraceae* family complexed with the MCAA and MCPA intermediate complexes, confirms the catalytic water molecule is positioned for potential deprotonation by His^183^ and appears to be activated by a His/Glu dyad, which is not present in other l-HADs [108].

Structural studies on *Saccharomyces cerevisiae* haloacid dehalogenase reveals an interesting insight into the molecular basis of their substrate specificity as it contrasts with other organisms. The biochemical promiscuity of the phosphatases haloacid dehalogenase with their structural flexibility and catalytic efficiency indicates the evolution of substrate specificity does not necessarily follow sequence divergence. Although the variation of enzyme substrate preferences within several families of HADs from yeast can convergently evolve to catalyze the dehalogenation of the same substrate [59].

Structural features that specifically confer defluorinating activity on fluoroacetate dehalogenase (FAcD) to break the carbon-fluorine bond differs entirely from other haloalkane dehalogenase despite their shared parallel mechanisms of dehalogenating ejection of halide anion by a nucleophilic attack of the substrate. The molecular basis of the biocatalytic defluorination requires the close and most precise placement of the binding residues to effectively stabilize the small fluoride ion [72].

### 1.4. Protein Engineering in Dehalogenases

The application of protein engineering tools in mutagenesis studies and sequence analysis indicates that several dehalogenases are homologous to enzymes that carry out transformations on halogenated and non-halogenated substrates. More so, construction of heterologous expression systems, modification of reaction mechanisms, and the purification to homogeneity to the structural resolution of the dehalogenase enzymes are the hallmark of the significant progress made in engineering metabolically active organohalogenated remediating enzymes [49,55,57,84,85,115,180,181,182,183].

The evolution of dehalogenase activities by the modification of existing hydrolase activities or decryptification of silent hydrolase genes in consort with acquisitive evolution can give rise to specific enzymic activities in response to environmental conditions. Thus, insights into the entire catalytic cycle of dehalogenases are necessary for the rational enzyme engineering [184]. To date, two steps of the catalytic cycle are described in some dehalogenases such as the HLDs. More so, mutational substitutions of catalytically active residues have shown that increase in enzyme rate can be facilitated by decreasing the number of interactions between the main and cap domains [185]. Extensive site-directed mutagenesis has been directed at elucidating the role of catalytic amino acid residues in dehalogenases catalytic mechanism [106,186]. Histidine and arginine amino acid residues mutational analysis are identified to play a part in the catalytic mechanism of dehalogenase as described in *Pseudomonas cepacia* MBA4 and hydrolysis of the alkyl-enzyme intermediate of *Xanthobacter autotrophicus* GJ10 (Dhla) [55,183]. Comparison analysis by Chan et al. [72] of the active site dehalogenase suggests that motional freedom of histidine residue is insufficient sometimes to yield additional space in the halide pocket during halide selectivity. Directed evolution of the structural dehalogenase gene HDL IVa has shown apparent implication of these residues in the activity of the 2-haloacid halohydrolase IVa dehalogenase from *Pseudomonas cepacia* MBA4 [187]. Rational design and the substitution of the Asp^260^ to asparagine in a haloalkane dehalogenase gene (DhlA) resulted in reduced activity towards all brominated substrates tested. Although mutation of Asn^148^ (analogous to the catalytic aspartate), with aspartic or glutamic acid restored activity with reduction of the rate of carbon-bromine bond cleavage and the rate of hydrolysis of the alkyl-enzyme intermediate [86].

Improvement in expression and solubility of haloalkane dehalogenase has been achieved through the rational design engineering and molecular evolution of a HaloTag7 (a catalytically inactive derivative of DhaA) to rapidly form a covalent attachment to synthetic chloroalkane ligands when fused to a protein partner [188]. Active site residues provide inactivation protection to some dehalogenase enzymes; however, the substitution of these conserved residues could significantly affect enzyme activity. The nonproductive binding of some small halocarbons such as 1,2-DCE in some DhlA due to the inability to displace water and halide molecules from the active site could be targeted by Site-directed mutagenesis and directed evolution experiments to the cap domain as the target region for engineering [189]. Detailed exploration of the enzyme substrate and enzyme-product complexes revealed the possible importance of the active site waters and halide ions for binding of small ligands in the active site.

The lower activity of some dehalogenase towards larger halogenated compounds is likely caused by the increased energy barrier required to overcome the steric hindrance, however, quantum studies have shown evidence of the electrostatic influence of two active-site waters on the rate-limiting barrier of 4-chlorobenzoyl-CoA dehalogenase [190]. Depending on the structure dynamics of the fluoroacetate dehalogenase, site-directed mutagenesis has shown how the supply of hydrogen bond to the halide ion by the catalytically active residues is important in the reduction of the activation energy for the cleavage of the carbon-fluorine bond [191]. The surface hydrophobicity is important towards the thermostability of dehalogenases, and by in silico design strategy and directed multiple mutations of these hydrophobic surface residues, thermostability can be enhanced [192]. Site-directed mutagenesis has shown conserved Aspartate residues not required for tight substrate/product binding in 4-chlorobenzoyl-coenzyme A (4-CBA-CoA) dehalogenase to be essential for the enzyme activity. In events of chemical modification, the active site tryptophan residues play a major role in preventing loss of activity. The AtzA genes obtained from *Aminobacter aminovorans* isolates displayed an evolutionary accumulated variety of mutations acquired by natural selection which conferred changes in substrate specificity for halide ion [193]. Both homology modeling and site-directed mutagenesis of two residues within the binding pocket geometry with consequent alterations at residue positions Ile^253^ and Gly^255^ improved the rigidity of the substrate binding pocket in fine-tuning the enzyme specificity [175,194]. Combinatorial randomization within the binding pocket geometry has also improved the catalytic efficiency 20-fold greater than the wild-type AtzA [195]. Single substitution at Val^12^ and Leu^395^, respectively and random mutations (Met^315^, His^399^, Asn^429^, and Val^466^) via directed evolution have been demonstrated to improve *Aminobacter aminovorans* AtzA dehalogenase variants catalytic efficiency [196]. Mutational analysis of active site mutant of atrazine chlorohydrolase (TrzN) describes the mechanistic role of threonine in the catalytic efficiency of the enzyme to be reminiscent of carbonic anhydrase, in which the threonine positions water molecule for reaction with carbon dioxide [197].

Genetic programming of dehalogenase-producing bacteria can endow such engineered strains with expanded biochemical activity towards biodegradation of halogenated compounds as described in a *Pseudomonas putida* biofilm production under the tight control of a cyclohexanone-responsive expression system [128]. Li et al. [198] turned a whale myoglobin into a functional nitric oxide reductase by modifying the pocket in the enzyme with several point mutations to allow binding of iron ion. Most haloacid dehalogenases share about 25 strictly catalytically important conserved charged and/or polar amino acid residues, and mutational reports on the indispensability of these residues to both activity and protein integrity have been demonstrated in varying ways [56,186]. In a way that suggests the indispensability of these residues in catalysis and protein integrity, the dehalogenting activity can be enhanced or inhibited as a consequence of such mutational changes [56,57]. Histidine has been revealed by site-directed mutagenesis to be inessential for catalysis in the haloacid dehalogenase hence hydrolysis of an ester intermediate is dependent on a different set of catalytic residues [23,106].

Analysis by chemical modification, site-directed mutagenesis as well as substrate modification has revealed a different substrate recognition mechanisms of Group I αHA dehalogenases which dehalogenates d,l-haloacids without formation of an ester intermediate state. Mechanistically, hydrolytic water performs a direct nucleophilic attack on the substrate, whereby solvent water molecule activated by a catalytic base directly attacks the α-carbon of the substrate to release a halide ion [50,99]. This phenomenon was first demonstrated in enzymatic hydrolytic dehalogenation experiment of d,l-2-haloacid dehalogenase from *Pseudomonas* Sp. 113 [51]. Enzyme isomerization step at prevailing higher halide concentrations induces conformational changes in the cap domain that is necessary to the solvation of the halide ion by the water molecule [199]. Mutational studies on ester intermediates of mesophilic haloacids l-DEX-YL and DhlB dehalogenase have previously identified Ser^118^ as the binding residue for the carboxylic acid group. In DhlB and the *S. tokodaii* dehalogenase, Ser^114^ and Ser^95^ perform the job, respectively [22,107]. The substrate binding residues of non-stereospecific α-haloalkanoic acid dehalogenase can be fine-tuned towards halogenation of haloacids. The specificity of this enzyme design, particularly at the S188V residue position, has facilitated the interconversion of β-halogenated compounds such as a 3-chloropropionic acid (3CP) of an α-haloalkanoic acid dehalogenase E (DehE) from *Rhizobium* Sp. RC1 [200]. The single active site cavity of this enzyme permits the binding of 3CP, as well as the α-chlorinated acid compounds D-2CP and l-2CP, at residues Trp^34^, Phe^37^, and Ser^188^ computationally [201]. Site-specific mutagenesis of functionally important active site residues indicates that the dehalogenting mechanisms of the *Rhizobium* Sp. dehalogenase DehE are non-stereospecific [202].

The dynamics of the substrate export routes are constrained in the upper tunnel of most dehalogenases by the side-chain of Cys^176^ which significantly influences the size and shape of their entrance tunnels. Directed evolutionary design demonstrated the global effect this residue position can have on the active-site structure in a Cys-176Tyr-DhaA mutant [203]. Furthermore, this mutation allows a more productive binding of 1,2,3-trichloropropane (TCP) within the active site, which when further fine-tuned by Tyr273Phe in a random mutagenesis and genetic engineering of a chloropropanol-utilizing bacterium, can create a possible modification of protein access and export routes for enhanced catalytic activity towards halogenated substrates [204]. The access tunnels play an important role in substrate specificity, catalytic activity and enantioselectivity. For example, site-directed mutagenesis of specific entrance tunnel residues identified by structural and phylogenetic analyses has generally increase catalytic activity and substrate specificities of mutant enzymes [71]. Narrowing the access tunnel of dehalogenase by rational design and directed evolution of residues in access tunnels in the buried active site not only results in a preference for small substrates but also successfully enhanced degradation of TCP by decreasing accessibility of the active site for water molecules, thereby promoting activated complex formation. The mutations also improved carbon-halogen bond cleavage and shifted the rate-limiting step [205]. Stability to organic cosolvents by dehalogenases could be achieved through modification of access tunnel rigidity as reported in a haloalkane dehalogenase DhaA from *Rhodococcus rhodochrous* NCIMB 13064 [14]. In enzyme-solvent interactions of dehalogenases, solvent molecules may not act as competitive inhibitors, but Molecular Dynamics (MD) simulations with other validation have been used to show how cosolvent molecules can be trafficked into the enzymes’ access tunnels and active sites, which enlarges their volumes with no change in overall protein structure [206,207].

Evolutionary processes targeting predefined sequence space in the generation of deletions and repeats are most successful for haloalkane dehalogenases considering their substrate specificity. Truncation by random combinatorial fusion in some dehalogenase is of substantial importance in their substrate adaptation, the stability of the transition state and cap domain restructuring [64,182]. Expanding substrate adaptation can profit from site-directed mutagenesis [100], and more so, the flexible residues of the cap and main domain can be the target of enhancing the catalytic properties of dehalogenases. Molecular simulations have shown how flexible regions of the cap domain engineered with connecting disulphide bridges can confer substantial thermal and desaturating stability [64].

### 1.5. Screening and Prospecting of Dehalogenase in Microbes

Continued efforts towards the discovery, isolation and characterization of new dehalogenating microbial species can be seen as an important way of mobilizing adaptation in microorganisms in the biodegradation of xenobiotics. Efforts in facilitating the prospecting and the discovery of novel dehalogenases with potential for broader applicability has paid off with more challenges encountered as the recalcitrance of these dehalogenated compounds persist. Several approaches including sequence- and activity-based screening are a reliable determinant in accelerating the discovery of novel dehalogenases with improved or modified activities [82]. A bacterial enzyme that presumably evolves from a halogenated polluted environment can be well suited for transforming other halogenated contaminated environments [134,208]. Therefore, experimental enrichment of growth conditions with the carbon and nitrogen requirements for the screening of dehalogenase producing microbes can be implemented. Anaerobic microbial enrichment culture can be used where reductive dehalogenation of trifluoroacetic acid and a net loss of hydrogen measurement from anoxic growth media dosed with various halogenated compounds can be of profit in the prospect of *Dehalococcoides* spp [42,209,210].

Other dehalogenase-producing strains like the native bacterium strain MFA1, which belongs to the Synergistetes phylum of the Australian bovine rumen have been isolated using anaerobic agar plates with amino acids metabolized in the presence of fluoroacetate as a carbon source [80,211]. Few soil microorganisms that can utilize short-chain haloalkanoic acids such as chloroacetate and 2-chloropropionate as their sole carbon source have evolved over time [92]. But these Substrates can incorporate radioactivity into the enzyme when used in enrichment culture media for screening certain fluoroacetate dehalogenase bacteria species [81,148]. Although fluoroacetate as the sole carbon source can mediate production of fluoroacetate dehalogenase (FAc-DEX FA1) [74], the presence of ammonia in the substrate mixtures will modify the active-site carboxylate groups to inactivate the enzyme [118]. Utilizing fluoroacetate as terminal electron acceptor rather than a carbon source makes enrichment culture media suitable for growth screening via the reductive dehalogenation pathway [211].

The *Rhizobium* spp. are known to utilize a wide range of carbon sources for growth. Elective culture media of chlorinated aliphatic acids have been used in the isolation of *Rhizobium* Sp. producing dehalogenase [119,212]. The ability of *Rhizobium* Sp. RC1 to utilize a variety of non-halogenated carbon sources have been examined to show that isolation and maintenance of this bacterium can best be achieved with growth on pyruvate, lactate, acetate, mannitol, glycerol, glucose, ribose, sucrose and serine. Additionally, *Rhizobium* Sp. RC1 uses as sole sources of carbon and energy in minimal media 2,2-dichloropropionate, d,l-2-chloropropionate to support growth. In a way that is consistent with the nutrient requirement for their growth, *Pseudomonas putida* stains and *Alcaligenes xylosoxidans* can grow on enriched media by continuous-flow enrichment culture with 22DCPA as the sole carbon and energy source [213,214]. To avoid thermal dechlorination, the *Pseudomonas putida* strains can be maintained on mineral salts medium supplemented with sodium succinate and with either 2MCPA or 22DCPA as the sole carbon and energy source [213,215].

Minimal salts both in solid and liquid enrichment media have been useful in isolating dehalogenase-producing *Anthrobacter* spp. strains. The α/β-halocarboxylic acids [2,2-dichloro-propionic acid (2,2-DCP), d,l-2-chloropropionic acid (d,l-2-CP), d-2-chloropropionic acid (d-2-CP), l-2-chloropropionic acid (l-2-CP), 3-chloropropionic acid (3CP), monochloroacetate (MCA), dichloroacetate (DCA), and trichloroacetate (TCA)] as well as other basal salts can be utilized in the growth of such microbes [116]. Enrichment medium supplemented with 2-chloropropionic acid have been adapted in the combined qualitative pH indicator and quantitative HPLC method in the isolation of *Paracoccus* Sp. DEH99 2-haloacid dehalogenase-producing bacteria [216].

Bacteria affiliated to the Gram-positive and Gram-negative genera can be isolated through strategies involving direct plating or liquid batch cultures in aerobic or anaerobic conditions. Minimally defined aerobic basal (mDAB) media supplemented with vitamins and anaerobic media under O_2_ free N_2_. Dalapon (2,2-dichloropropionic acid) and 2MCPA (2-chloropropionic acid) have been used in the isolation of novel bacteria of the *Proteobacteria* and the Gram-Positive *Bacillus* and *Enterococcus* genera able to degrade α-halocarboxylic acids [217].

Vinyl chloride dechlorinating enrichment culture and sediment-free culture derived from tetrachloroethene PCE-to-ethene-dechlorinating microcosms when enriched with acetate as the electron donor can be used in the sequential transfers of cultures from PCE-to-ethene-dechlorinating microcosms, in a defined bicarbonate-buffered mineral salts medium. This, when reduced by amended lactate, pyruvate and vinyl chloride can yield an ethene-producing enrichment culture suitable for the identification of *Dehalococcoides* spp [218]. The entire process of preparing the enrichment media dilutes any methanogenic archaea that may affect the enrichment processes. Circumstantial evidence indicates that type of enrichment culture desirable for obtaining *Dehalococcoides* spp. is important. Care must be taken in habitat prospecting especially many chloroethene-contaminated sites should be sort. Because VC is not used as metabolic electron acceptor but rather growth supporting electron acceptor, and co-metabolic VC reduction requires the presence of higher chlorinated ethenes which is significant in the physiological characteristics of *D. ethenogenes*-type populations [219].

Inducible dehalogenation process can be influenced sometimes only in a coculture environment. The dehalogenating process often requires the presence of both dehalogenating and non-dehalogenating microorganisms. During the reductive dechlorination of hexachlorocyclohexanes (HCHs) contaminated soil, the anaerobic metabolic dechlorination by a *Dehalobacter* Sp. was inducible in the presence of a non-dehalogenating *Sedimentibacter* Sp. [220]. Coenzymes are important in catalyzing the removal of a halogen atom from the unsaturated aliphatic organohalogen compound by the addition of a water molecule to the substrate. New forms of dehalogenases require the reduced form of flavin adenine dinucleotide (FAD) for a spontaneous hydration of unsaturated organohalides into their corresponding products [138]. Whereas, the NADPH is required as a co-substrate for the asymmetric reduction of 2-chloroacrylic acid to yield (*S*)-2-chloropropionic acid [139]. Defined synthetic mineral medium without any complex additions and with pyruvate as the carbon and energy source has been utilized in a bacterial mixed culture that dehalogenates trichlorobenzenes [221].

### 1.6. Metal Co-Factor-Dependent Dehalogenases

Several dehalogenases have been described on the basis of their natural hydrolytic halogenating property [10,11]. However, enzymes incorporated with cofactor metal sites have been elaborated to enhance the functionality, better performance, and/or novel functions of designed enzymes as mimics for metalloenzymes [222]. Metalloenzymes are found in all enzymes families, however simple classification of metal-containing subgroups is based upon the bioinorganic motifs [223]. Proteins evolved together with the metal ion abundantly present in Nature, and the metal ion provides diversity for the function and structure of protein [224,225]. The role of metal ion in protein is mostly divided into structural or enzymatic [226,227,228]. Zinc fingers are one of the popular representatives on how the metal can provide stability and affect the folding of the protein, though folding of the small protein into various ways with different metal binding residues using the same zinc ion depends on the peptide sequences [229]. In addition to maintaining the protein fold, the metal also controls overall structural stability which is affected by pH, temperature and other physical conditions [230]. 

Native metallo-dehalogenases are rarely found although several dehalogenases are metal-dependent and it is difficult to identify adaptations to an enzyme that enhance its ability to dehalogenate a xenobiotic substrate. Therefore, since protein metal-binding sites are responsible for catalyzing some of the most difficult and yet important functions [10], this knowledge is utilized in designing metallo-dehalogenase with reproducible structural and functional features as native metalloproteins. The catalytic activity of some dehalogenase is metal ion-dependent as established in the chlorothalonil hydrolytic dehalogenase (Chd) [149]. Chlorohydrolases that act on haloaliphatic and halobenzene substrates differ mechanistically from other well studied halohydrolases which require water as a cosubstrate to catalyze an overall hydrolytic displacement reaction [10]. The metallo-dehalogenase enzyme; atrazine chlorohydrolase (AtzA, AtzB, AtzC & TrzN) from *Pseudomonas* Sp. ADP is known to have substoichiometric quantities of transition metals and divalent transition-metal ion dependent. These structurally defined metallo dehalogenases; atrazine chlorohydrolases AtzA and TrzN, catalyzes hydrolytic dechlorination reaction via a hydrolytic mechanism dependent on divalent metal ion (Fe^2+^ and Zn^2+^), respectively [197,231]. The TrzN chlorohydrolase plays an alternate AtzA role in other Gram-positive bacteria *Arthrobacter* and *Nocardioides* [176]. Similarly, the binding domain residues orientation does not permit nucleophilic substitution by an activated water molecule in AtzA, while the histidine in TrzN, establishes a hydrogen bond to the water molecule coordinated with the zinc ion [175]. With less native metal-dehalogenase, artificial metallo-dehalogenases can be designed based on the active-site metal centre optimization to yield promiscuous catalytic activities which could expand the catalytic repertoire of the dehalogenases with higher stability, greater efficiency, or even unprecedented non-natural functions.

Recent successful design of artificial metalloenzymes using the computational approach as the first step has enabled the prediction of designed protein with established functionality in practical attempts of enzyme characterization [198,232]. A wide range of metal ions have demonstrated their utility in enhancing dehalogenase transformation of halogenated compounds, and knowledge of the critical factors that govern catalytic efficiency and other properties allows researchers to begin incorporating metal cofactors to pursue better performance or novel functions. Engineered chlorothalonil hydrolytic dehalogenase (Chd) having a binuclear Zn^2+^-Zn^2+^ centre when substituted with other divalent cations, such as cobalt and cadmium, and manganese and calcium showed higher catalytic efficiencies of chlorothalonil dehalogenation [149]. Thus, by grafting in a metal binding site into the active site of dehalogenases, and designing robust metal coordination in these enzymes accelerated scope of their functionality can be optimized.

Quite a few members of the dehalogenase family of enzymes lack metal binding domains as demonstrated in the structures of 4-chlorobenzoyl CoA dehalogenase, and l-2-haloacid dehalogenase [22,112]. The TCE-RDase is a peripheral membrane-bound protein of the cytoplasmic membrane in the dehalorespiratory electron transport chain of *D. ethenogenes*. It has been characterized to contain cobalamin and iron-Sulphur clusters just as the halocarbon RDases class of enzymes [122]. The redox cofactors harboured by these microbes and others found in the sequence of other periplasmic or cytoplasmic membrane proteins act as a common export pathway [233].

### 1.7. Creating an Artificial Metal Binding Site

Creating artificial metalloproteins poses a lot of challenges as a single mutation on protein will cause the protein to be destabilized. In this era of highly advanced computational technology, scientists are now working on protein engineering using computational tools to first study the variability. Parmar et al. [234], have concluded that the design of artificial metalloproteins can be divided into three types, creating new artificial metal binding site into existing protein with known 3D structures, de novo design of new artificial metalloproteins harnessing the power of symmetry and designing a new metal binding site into flexible regions of proteins. Computational programs have been designed to search for the suitable potential metal binding site by putting the score for the suitable adjacent residues in the structure that can be mutated to form metal binding residues. Although the search algorithm may be different DEZYMER developed by Hellinga and Richards [235], METAL-SEARCH by Clarke and Yuan [236] and ROSETTA by Baker and Tezcan lab are suitable programs to select suitable residues to build an artificial metal binding site. Both DEZYMER and METAL-SEARCH do not alter the protein backbone but only mutate the side chain of an amino acid residue in a specific region search through metal binding geometry analysis. Symmetry de novo design of metalloproteins allows better allocation of metal to the design multimers of peptides. Based on the available symmetrical protein templates, the specific residue can be modified to metal binding residues once the in silico mutation prove the geometry is suitable for metal binding. In contrast to the computational design, design of an artificial metal binding site into flexible regions of proteins prove to be rather challenging as most artificial metalloproteins pick template containing potential metal binding residues located at the rigid backbone which mostly in secondary structure or located at fixed turn between secondary structure [234].

### 1.8. Potential Applications of Dehalogenases

Dehalogenases can be applied to different fields and their applications are still expanding. They have potential applications in bioremediation [237], biosensing [5], designing antidotes for warfare agents [238,239], synthesis of optically pure compounds [240,241], cellular imaging, and protein analysis [188,242]. The properties have been harnessed in the fields of industry, pharmaceutical, environment and green chemistry and are also described here;

#### 1.8.1. Application in the Construction of Expression Cassettes

The halide resistance genes can be used as selection markers for direct selection of constructs or transformants. Auxotrophic and phototrophic markers have been used in *Saccharomyces cerevisiae* genetics, but there are several auxotrophic markers that cannot be introduced easily because of their recessive nature and the polyploidy of many of these strains. Under the control of ADH1, CYC1 and GPD1 promoters, expression system constructs of the *S. cerevisiae* SFA1 and *Moraxella* Sp. dehH1 gene, encoding haloacetate dehydrogenase and formaldehyde dehydrogenase, respectively [79,243] can be used for direct selection of yeast strains based on resistance against either formaldehyde or fluoroacetate [147]. Dehalogenase genes from *Rhodococcus* spp. have been used as selection markers in *Escherichia coli* systems in the growth study and degradation of 3-chloropopionic acid [244].

#### 1.8.2. Application in the Production of Useful Compounds

The reaction mechanisms catalyzed by l-2-haloacid dehalogenase (l-DEX) and fluoroacetate dehalogenase does not involve a direct nucleophilic attack of the substrate by the solvent water molecule to displace the halogen atom [49,81], which is a setback in the production of some compounds. However, the nucleophilic attack of substrates by d,l-DEX 312 and other related dehalogenases is consequentially important in the production of industrially useful compounds other than 2-hydroxyalkanoic acids by employing a nucleophile other than a water molecule in the reaction process [142]. The 2-haloacrylate reductase dehalogenase can be useful in the production of chiral compounds in herbicides by the reduction of a carbon-carbon double bond of unsaturated organohalogen compounds such as 2-chloropropionate in the synthesis of aryloxyphenoxypropionic acid herbicides, which are some of the most abundantly used herbicides in the world [139]. The production of amino acids from 2-haloalkanoic acids with d,l-DEX 312 dehalogenase has also been attempted [142]. The 2-haloacid dehalogenases are useful for the production of optically active hydroxy acids, which are used for the synthesis of various pharmaceuticals and agrochemicals [49]. Similarly, stereoselectivity of 2-haloacid dehalogenases can be used to selectively dehalogenate one of the isomers of 2-CPA from its racemic mixture to generate a chiral reagent useful in the synthesis of herbicides and pharmaceuticals [245].

#### 1.8.3. Applications in Bioremediation

Assessing and monitoring chloroethene-contaminated sites can rely on nucleic acid-based approaches targeting *Dehalococcoides* by 16S rRNA [219]. Dehalogenating routes vary with different dehalogenases, the reductase halogenase in different microorganisms reductively dechlorinates by mainly attacking the *meta-* and/or *para*-chlorines of PCB mixtures, especially in chloroethene-contaminated sites [246]. Bacterial mixed cultures of microbial consortium from the family microbial consortium phylogenetically affiliated with a sublineage within the *Desulfovibrionaceae* and the gamma subclass of *Proteobacteria* had been developed and used to reductively dechlorinate trichlorobenzenes [221]. The enrichment cultures of the *Dehalococcoides* and other microbial cultures of *Desulfitobacterium* spp. and *Sulfurospirillum multivorans* have been used in the depletion monitoring of heavy isotopes in halogenated products as determined by the extent and mechanisms of carbon isotope fractionation during reductive dehalogenation [84,85]. Association of dehalogenases in a preindustrial gene pool among these microbes provides an evolutionary precursor genetic adaptation to catalyze dehalogenation of naturally occurring halogenated compounds. The role and physiological diversity in degradation of chlorinated organic contaminants and the traits of this interesting group of microorganisms have been given a detailed review [150].

In the LinB-enzyme catalyzed transformation of hexachlorocyclohexane (HCH)-contaminated environments, a similar degradation pathway has been demonstrated to be evolved by the *Sphingobium indicum* B90A LinB dehalogenase in the biotransformation of stereoisomeric mixtures of HBCDs. Although the enzymatic transformations are typical of a mixed order, the rate order kinetics can be affected by the substrate and binding variability [13]. Consequently, various hydroxylated metabolites or intermediates formed from either initial steps or hydrolytic dehalogentaion of HCHs could pose serious and unknown environmental risks [135].

The haloalkane dehalogenases have been exploited for microbiological detection and removal of the halogenated by-products in chemical synthesis and halocarbon pollutants in the environment [184]. Mena-Benitez [247], engineered a tobacco plant by introducing *DhlA* (haloalkane dehalogenase) and *DhlB* (haloacid dehalogenase) to degrade 1,2-dichloroethane[14]. In combination with the endogenous alcohol dehydrogenase (ADH) and aldehyde dehydrogenase (ALDH), a new metabolic pathway was created that can convert 1,2-dichloroethane directly into glycolate.

#### 1.8.4. Applications in Drinking Water Treatment

In spite of their importance to public health and their prominence in drinking water, most halogenated compounds can be biodegraded by dehalogenase producing organisms, playing beneficial roles in suppressing the concentrations of organohalides in drinking water treatment and in drinking water distribution systems. In fact, haloalcohol depletion in some drinking water distribution systems has been observed and attributed to dehalogenase biodegradation [248,249]. Studies have suggested that *Afipia* spp. have a beneficial role in suppressing the concentrations of haloacetic acids in tap water [250]. Large-scale of groundwater purification to remove 1,2-dichloroethane using *Xanthobacter autotrophicus* GJ 10 through treatment plants has been carried out and good results obtained [237].

#### 1.8.5. Applications in Detoxification

Ingestion of fluoroacetate-producing plants is believed to be toxic to grazing herbivores. Ingestion by livestock of such plants often results in fatal poisonings, which causes significant economic problems to commercial farmers in many countries. Production of a strong inhibitor (2*R*,3*R*)-erythro-2-fluorocitrate, of aconitase of the citric acid cycle during citrate synthase conversion of fluoroacetate is partly responsible for this toxicity [251]. Genetically modified fluoroacetate dehalogenase-producing bacteria capable of degrading fluoroacetate have been developed to protect ruminants from fluoroacetate toxicity. The fluoroacetate dehalogenase enzymes identified in some of these bacteria appear to degrade fluoroacetate, where an ester is produced as an intermediate which is hydrolyzed by a water molecule to form glycolate [80].

#### 1.8.6. Applications in Decontamination

Dehalogenases such as the haloalkane dehalogenases (HLDs) also play a role in decontamination of warfare agent such as sulphur mustard. With suitable HLDs, sulfur mustard can directly enter the active site and be converted to non-toxic thiodiglycol sulfoxide. Without HLDs, sulfonium ion is formed during spontaneous hydrolysis of sulfur mustard, this ion can cause massive cellular damage [239]. A specially engineered HLDs that is used to degrade sulfur mustard, called Yperzyme, has been developed and launched by Enantis, a biotechnology company based in the Czech Republic. This enzyme can be used to clean up large stockpiles of old stocks of sulphur mustard [238].

#### 1.8.7. Applications in Biosensing

The need for devices capable of measuring water contaminant concentrations in situ has led to the use of haloalkane dehalogenases in biosensor development. With the ability of HLDs to catalyze the conversion of different types of halogenated compounds into alcohol, halide ions and proton; the concentration of halide ions or protons then can be measured by ion selective transducer/pH meter. Whole cell biosensor can be developed with the dehalogenase as a biocomponent of fibre optic biosensor as demonstrated in DhlA of whole cells of *Xanthobacter autotrophicus* GJ10, and in fluorescence pH biosensor [5,14,185]. These are being created over time and improved from time to time to increase the sensitivity, reduce reaction times and improve the selectivity for halogenated compounds. The traditional way of sampling water using chromatography is time consuming and expensive, so the development of biosensors to detect the presence of a halogenated compound in situ surely will allow drinking water to be sampled and its quality to be under control. The dehalogenases have also been used in the development of covalent tethering of organic probes for cell imaging and protein analysis based on the ability to specifically label proteins with a wide range of optical properties and functionalities thus obtaining valuable reporting on a protein expressed in live cells [188,242].

### 1.9. Other Persistent Organic Pollutant (POPS) of PCDD/Fs

Emissions from combustion or various industrial processes result in the accumulation of sediments which constitute an important reservoir of polychlorinated dibenzo-*p*-dioxins and dibenzofurans (PCDD/Fs). Enriching for microbial reductive dechlorination of these polychlorinated pollutants by a biological process that may transform PCDD/Fs and potentially decrease their toxicity through the removal of lateral chlorines is important for assessing the potential for remediation of these contaminated sediments [252,253,254,255]. More so, their limited bioavailability in the environment and their overall chemical stability makes dechlorination of PCDD/Fs often too slow to significantly impact ambient concentrations of PCBs in the sediments column [256]. Under anaerobic conditions, the use of alternate halogenated co-substrates enhances the dechlorinating potential of indigenous microorganisms [257,258]. In the dechlorination of PCDD/Fs, the most prominent *Dehalococcoides ethenogenes* strains contain multiple genes predicted to encode for enzymes mediating reductive dehalogenation. They employ different dechlorination routes because of differences in their diverse metabolic activities in response to specific environmental contaminants or enrichment by co-substrates to dechlorinate CDD/Fs [259,260,261,262].

Despite the abundance of these dechlorinating bacteria, examining the congener profiles of historical PCDD/Fs by indigenous microbial dechlorination suggest the potential for anaerobic microbial dechlorination of weathered PCDD/Fs could still be unsubstantial, suggesting that the recalcitrance of aged PCDD/Fs in sediments could persist due to the limited bioavailability of PCDD/Fs because of these compounds being tightly sorbed in the sediments [263].

Anaerobic bacteria use one of a few pathways for the transformation and removal of the most abundant PCDD/F congener in the environment without destroying the PCB backbone. In this case, PCB mass is decreased producing lightly chlorinated congeners typically lower in toxicity and more amenable to aerobic degradation and volatilization [268]. Concerns have been raised on the sources of most recalcitrant PCDD/Fs, particularly in the food industry, with implications of artificial high-intensity sweeteners in the release of PCDD/Fs under certain conditions as an instance of a health threat. Sucralose, a widely-used artificial sweetener in various food and beverages decomposes at higher temperature thereby participating in chlorination reactions with the release of highly toxic compounds. Under certain thermoanalytical techniques, sucralose is stable, however, thermal degradation of sucralose or foodstuffs in the presence of sucralose at high temperatures can result in the generation of toxic PCDD/Fs [269]. The implication of cooking utensils and rusts (metal oxides) on the formation of PCDD/Fs under these conditions have been demonstrated [270]. PCDD/Fs have been classified to belong to the group of the strongest poisons among the known chemical compounds and relevant limitations in their production, application, thermal combustion as well as strict requirements for the use of compounds with PCCD/Fs emission potential have been ratified by the Stockholm Convention (2001). Consequently, emission sources of the most important and known polychlorinated dibenzo-*p*-dioxins (PCDDs) and polychlorinated dibenzofurans (PCDFs), known methods of reduction of emission to the atmosphere, the mechanism of dioxin formation in thermal processes and minimizing their formation removal from the stream of waste gases have been reviewed [271]. Higher dichlorination of PCDD/Fs can also be achieved in ambient temperature conditions, in the presence of metals, solvents and under ionic potential when microbes are limited [266,272]. A wide range of compounds are capable of PCDD/Fs formation which could be a *de novo* synthesis in the macromolecular carbon structures from various carbon species with various activated carbon such as, bituminous coal, charcoal, and fly ash. Precursors of small organic molecules such as aliphatic compounds, monocyclic aromatic compounds without functional groups, monocyclic aromatic compounds with functional groups, chlorinated aromatic compounds, and anthraquinone derivatives are also used. Consequently, the mechanism follows the formation of a halide complex; ligand transfer of the halide to a carbon atom contained in a macromolecular structure, and breakdown of the macromolecule into small compounds [273]. In the non-microbial controlled pyrolysis of biomass, the isomer distributions of PCDD/Fs are more selective compared to those reported from wood burning and waste incineration. At relatively low temperature a preferred formation pathway of PCDFs involving (chloro)phenol precursors is favoured with sequential chlorination [274].

## 2. Conclusions

Organohalides are highly toxic to living beings due to their carcinogenic, mutagenic and cytotoxic properties. Several bacteria that use halogenated compounds as their sole carbon and energy sources have been isolated and characterized. The discovery, isolation and characterization of new dehalogenating microbial species can be seen as an important way of mobilizing adaptation in microorganisms in the biodegradation of organohalide pollutants. Efforts in facilitating the prospecting and the discovery of novel dehalogenases with potential for broader applicability has paid off with several approaches including protein engineering in accelerating the discovery of novel dehalogenases with improved or modified activities. Since more challenges are encountered as the recalcitrance of these dehalogenated compounds persist, directed evolutionary mutagenesis of the dehalogenase enzymes could liberate the enzymes from their evolutionary native state to a much more efficient catalyst. The evolutionary trapped metal ion abundantly present in nature provide diversity for the function and structure of native dehalogenase proteins. This natural hydrolytic halogenating property of dehalogenases can be utilized in designing metallo-dehalogenase with reproducible structural and functional features as native metalloproteins based on the metal ion dependence of their catalytic activity. Therefore, in the perspective of effectiveness of vast majority of the organohalide utilizing microbes with conferred adaptation to severe metabolic stress arising from the toxicity of organohalide compounds, construction of heterologous expression system, modification of reaction mechanisms, and the purification to homogeneity to structural resolution of the dehalogenase enzymes can be the hallmark of the significant progress in engineering metabolically active organohalogenated remediating dehalogenases.

## Figures and Tables

**Table 1:** Some microorganisms with their dehalogenation properties.

Organism	Dehalogenase Designate	Habitats	Substrate of Halogenation	Corresponding Product	Property of Reaction Mechanism	References
*Moraxella* Sp. strain B	haloacetate dehalogenase H-1 and H-2	soil	l-2-haloacid	d-2-hydroxy acids	stereospecific	[95]
*Pseudomonas* Sp. YL, *Pseudomonas putida* No. l09, *Pseudomonas* Sp. CBS3	l-2-haloacid dehalogenase, 2-haloacid dehalogenase	soil	l-2- chloropropionate, 2-monochloropropionate	lactate, glyoxylate, and pyruvate	stereospecific	[49,57,96]
*Pseudomonas cepacia* MBA4	l-2-haloalkanoic acid dehalogenase IVa	soil	monobromoacetic acid	N.S	stereospecific	[47]
*Xanthobacter autotrophicus* GJ10	haloacid dehalogenase (*dhlB*)	soil	2-halogenated carboxylic acids	d-Lactate	stereospecific	[53,97]
*Pseudomonas. putida* AJ1 (*hadL*)	l-2-haloakanoic acid halidohydrolase	soil	2-monochloropropionic acid	lactate with the release of chloride	stereospecific	[98]
*Pseudomonas* Sp. 113	d,l-2-haloacid dehalogenase		d- and l-2-haloalkanoic acids, producing	l- and d-2-hydroxyalkanoic acids	non-stereospecific	[51]
*Pseudomonas putida* strain PP3	α-haloacid dehalogenase DehI and DehII	soil	d- and l-2-haloalkanoic acids	l- and d-2-hydroxyalkanoic	stereospecific	[99]
*Microbacterium* Sp. strain, ITRC1	hydrolytic dehalogenase (linB) and dehydrogenase (linC)	soil	γ-pentachlorocyclohexen and a 2,5-dichloro-2,5-cyclohexadiene-1,4-diol	2,5-dichlorophenol (2,5-DCP)	non-stereospecific	[100]
*Agrobacterium tumefaciens* RS5	hydrolytic haloalkanoic acid dehalogenase (DhlS5II) and cryptic l-isomer-specific dehalogenase (DhlS5I)	soil	2,2-dichloropropionate (DCPA), chloroacetic acid (MCA), dichloroacetic acid (DCA), and 2-chloropropionic acid (CPA)	N.S	non-stereospecific and stereospecific	[101]
*Burkholderia cepacia* MBA4	dehalogenase IVa (*hdlIVa*)	soil	l-2-haloacid	d-2-hydroxyacids	stereospecific	[47,56]
*Pseudomonas pavonaceae*	*cis*- and *trans*-3-chloroacrylic acid dehalogenase (CaaD and cis-CaaD)	soil	*cis*- and *trans*-3-chloroacrylate	malonate semialdehyde	specific hydrolysis	[102]
*Burkholderia* Sp. WS	(*S*)-2-haloacid dehalogenase, 2-haloacrylate r	soil	(*S*)-2-haloalkanoic acids, 2-chloroacrylate	(*S*)-2-chloropropionate, (*R*)-lactate, (*S*)-2-chloropropionic	NADPH-dependent reduction	[103,104]
*Pseudomonas* Sp. Strain CBS3	4-chlorobenzoate dehalogenase	soil	4-chlorobenzoate	4-hydroxybenzoate	non-stereospecific	[105]
*Pseudomonas* Sp. strain YL	2-haloacrylate hydratase l-2-haloacid dehalogenase (l-DEX) (d,l-DEX)	soil	l-2-chloropropionate –chloroacrylate (2-CAA) of l and d isomers of 2-CPA of l-2-haloalkanoic acid, monochloroacetate and monoiodoacetate, as 2-bromohexadecanoate	2-chloro-2-hydroxypropionate, d- and l-lactates, d-2-hydroxyalkanoic acids	Stereo and non-stereospecific	[106]
*Methylobacterium* Sp. CPA1 (dl-DEX Mb)	d,l-2-Haloacid dehalogenase	soil	d- and l-2-haloalkanoic acids, d- and L-2-chloropropionates	l- and d-2-hydroxyalkanoic acids	non-stereospecific	[50]
*Sulfolobus tokodaii*	l-2-haloacid dehalogenase	soil	chloropropionic acid		stereospecific	[107]
*Marine Rhodobacteraceae*	l-haloacid dehalogenase	soil	monobromoacetic acid (100%) followed by monochloroacetic acid (MCAA) (71%), *S*-bromopropionic acid (71%), *S*-chloropropionic acid (MCPA) (10%) and dichloroacetic acid	N.S	stereospecific	[108]
*Burkholderia* Sp. FA1	fluroacetate dehalogenase	soil	fluoroacetate to glycolate	glycolate	non-stereospecific	[74]
*Rhodopseudomonas palustris* CGA009	reductive dehalogenase	soil	3-chlorobenzoate	3-chlorobenzoyl coenzyme A (3-chlorobenzoyl–CoA) to benzoyl-CoA and further to	non-stereospecific	[109]
*Rhodococcus* Sp. m15-3 (DhaA) and *Xanthobacter autotrophicus* GJ10 (DhlA)	haloalkane dehalogenase	soil	1,2-dichloroethane andtrihalopropanes to 2,3-dihalogenated propanols	2-chloroethanol, chloroacetaldehyde, chloroacetate, and glycolate	non-stereospecific	[110,111]
*Alcanivorax dieselolei* B-5	haloalkane Dehalogenase (DadB)	arctic Ocean	haloalkanes	alkanols	non-stereospecific	[112,113,114]
*Pseudomonas* Sp. strain 113	d,l-2-haloacid Dehalogenase,	soil	d - and l-2-chloropropionates, trichloroacetate	l- and d-lactates, oxalate	non-stereospecific dehalogenation	[59]
*Arthrobacter* Sp. strain TM-1	4-chlorobenzoyl-coenzyme A dehalogenase	soil	4-chlorobenzoyl coenzyme A (4-CBA-CoA), 4-chlorobenzoyl-CoA	4-hydroxybenzoyl coenzyme A (4-HBA-CoA), 4-hydroxybenzoyl-CoA	hydrolytic substitution	[115]
*Paracoccus* Sp. DEH99	2-haloacid dehalogenase	Marine sponge *H. perlevis*	2-CPA, 2-bromopropionic acid (2-BPA), and iodoacetic acid	chiral reagents	stereospecific dehalogenation	[116]
*Alcaligenes xylosoxidans* Sp. *denitrificans* ABIV	d,l-2-haloalkanoic acid halidohydrolase (DhlIV)	Soil	mono- and dichloroacetic acid and mono- and dichloropropionic acid	glycolate and pyruvate	specific hydrolysis	[117]
*Rhizobium* Sp.	haloalkanoate dehalogenases (DehL, (DehD, (DehE)))	soil	2,2-dichloropropionic acid, 2-chloropropionic acid, monochloroacetic acid, dichloroacetic acid, 2-chlorobutyric acid and 2,3-dichloropropionic acid	d(−) and l(+) lactate, pyruvate	stereo/non-stereospecific	[118,119,120]
*Methylobacterium* Sp. HJ1	2-haloalkanoic acid hydrolytic dehalogenase (DehE)	soil	2,2-dichloropropionic acid and d,l-2-chloro-propionic acid	to produce pyruvate and lactate	non-stereospecific	[121]
*Arthrobacter* Sp. strain S1						
*Dehalococcoides ethenogenes*	TCE reductive dehalogenase (TCE-RDase)		tetrachloroethene or trichloroethene (TCE)	ethene	non-stereospecific	[122]
*Dehalococcoides* Sp. Strain BAV1	reductive dehalogenase (RDase)	aquifer	chloroethene	ethene	non-stereospecific	[33,34,123,124]
*Sphingomonas chlorophenolica*	tetrachlorohydroquinone dehalogenase	soil	pentacholophenol	tetrachlorohydroquinone (TCHQ), trichlorohydroquinone, and 2,6-dichlorohydroquinone	non-stereospecific	[125]
*Shewanella sediminis*	reductive tetrachloroethene dehalogenase	soil	tetrachloroethene (PCE)	trichloroethene (TCE)	non-stereospecific	[126]
*Psychrobacter cryohalolentis* K5	haloalkane dehalogenase (DpcA)	Saline-water (Siberian permafrost	1b and other halogenated subtrates		non-stereospecific	[117,120]
*Sphingomonas paucimobilis UT26*	haloalkane dehalogenase LinB	soil	1,3,4,6-tetrachloro-1,4-cyclo- hexadiene to 2,5-dichloro-2,5-cyclohexadiene-1,4-diol via 2,4,5-trichloro-2,5-cyclohexane-1-ol during γ-HCH dechlorination	chlorophenols	non-stereospecific	[66,121,124]
*Agrobacterium radiobacter* strain AD1	haloalcohol dehalogenase (HheC)	soil	1,3-dichloro-2-propanol	eposide (chloride, halide and proton)	non-specific	[127]

N.S—Not specified.

**Table 2:** Some structurally resolved dehalogenases with their catalytic properties.

Organisms	Dehalogenase Complex	Gene	PDB Entry	Catalytically Active Residue	Halide-Stabilizing Residues	Refinement Resolution (Å)	Family	Reference
*Pseudomonas* Sp. YL	l-2-haloacid dehalogenase	l *-DEX YL*	1JUD	Asp-10, Asn-177 and Lys-151	Tyr-12, Asn-119, Lys-151, Asn-177 and Trp-179	2.5	homodimeric	[23]
*Pseudomonas* Sp. ADP	atrazine chlorohydrolase	*AtzA*	4v1x, 4v1y	Asp-327, Glu-246, His-243	N.S	2.2, 2.8	hexameric	[175]
*Xanthobacter autotrophicus*	haloalkane dehalogenase	*dhlA*	2DHC	Asp-124, His-289, Asp-260	Trp-125, Trp-175	1.9	α/β fold	[19]
*Rhodococcus* Sp.	haloalkane dehalogenase	*dhaA*	1BN6	Asp-117, His-283, Glu-141	Asn-52, Trp-118	1.5	monomer	[65]
Mycobacterium *tuberculosis* H37Rv	haloalkane dehalogenase	*Rv2579*	2QVB	Asp-109, His-273, Glu-133	Asn-39, Trp-110	1.19	monomer	[117]
*Bradyrhizobium japonicum*	haloalkane dehalogenase	*dhaA*	3A2M	Asp-103, His-280, Glu-127	Asn-38, Trp-104	1.84	homodimer	[120]
*Marine microbial consortium*	haloalkane dehalogenase	*dmmA*	3U1T	Asp-144, His-315, Glu-168	Asn-78, Trp-145	2.2	monomer	[21]
*Sphingobium* Sp. MI1205	haloalkane dehalogenase	*linB*	4H77	Asp-108, His-272, Glu-132	Asn-38, Trp-109	1.6	monomer	[121]
*Agrobacterium tumefaciens* C58	haloalkane dehalogenase	*datA*	3WI7	Asp-108, His-274, Glu-132	Asn-43, Tyr-109	1.7	monomer	[124]
*Bradyrhizobium elkani* USDA94	haloalkane dehalogenase	*dbeA*	4K2A	Asp-103, His-271, Glu-127	Asn-38, Trp-104	2.2	homodimer	[125]
*Bradyhizobium japonicum* USDA110	haloalkane dehalogenase	*dbjA*	3A2N	Asp-103, His-280, Glu-127	Asn-38, Trp-104	1.89	homodimer	[120]
*Plesiocystis pacifica* SIR-1		*dppA*	2XT0	Asp-123, His-178, Asp-249	Trp-124, Trp-163			[264]
*Strongylocentrotus purpuratus*		*dspA*	NA	Asp-120, His-285, Glu-144	Asn-53, Trp-121	N.S	N.S	[265]
*Alcanivorax dieselolei* B-5		*dadB*	NA	Asp-108, His-271, Glu-132	Asn-37, Trp-109	N.S	N.S	[112]
*Pseudomonas* Sp. YL	l-2-haloacid dehalogenase complexed with monochloroacetate, l-2-chlorobutyrate, l-2-chloro-3-methylbutyrate, or l-2-chloro-4-methylvalerate	l *-DEX*	1ZRN, 1ZRM	Asp-10	Arg-41	1.83, 2.0, 2.2, 2.2,	homodimer	[104]
*Burkholderia cepacia*	haloacid dehalogenase, l-2-monochloropropanoate intermediate	*DehIVa*	2NO4, 2NO5	Asp11 (Asp108), Ser119 and Asp181	Arg42 (Arg41, 39), Asn120 (Asn119, 115), Trp180 (Trp 179, Phe175)	1.93, 2.7	homodimer	[54]
*Rhodopseudomonas palustris*	fluoroacetate dehalogenases	*RPA1163*	3R3U, 3R3V, 3R3W, 3R3X, 3R3Y, 3R3Z, 3R40, 3R41	Asp110, His280, Asp134	His155, Trp156 and Tyr219	1.6, 1.5, 1.6, 1.8, 1.15, 1.7, 1.05, 1.05, 1.05	homodimeric	[266]
*Methylobacterium* Sp. CPA1	D,l-2-haloacid dehalogenase		4N2X	N.S	N.S	1.7	hexamer	[179]
*Pseudomonas putida* strain PP3	group I α-haloacid dehalogenase	*Dehl*	3BJX	(Thr-62, Glu-66), and Asp189	N.S	2.3	homodimer	[267]
*Sphingomonas paucimobilis* UT26	haloalkane dehalogenase	*LinB*	1MJ5	Asp-108, His-272, and Glu-132	Asn-38 and Trp-109	0.95	monomer	[12]
*Sphingomonas paucimobilis* UT26	haloalkane dehalogenase and complexes of linb with 1,2-propanediol/l-bromopropane-2-ol and 2-bromo-2-propene-l-ol	*LinB*	1K6E, 1K63	Asp-108, Glu-132, and His-272	Primary (Asn-38 and Trp-109), Secondary (Trp-207, Pro-208, and Ile-211)	1.85	monomer	[177]
*Sphingomonas paucimobilis* UT26	haloalkane dehalogenase LinB/LinB with 1,3-propanediol	*LinB*	1CV2, 1D07	Asp-108, His-272, and Glu-132	Asn-38 and Trp-109	1.58 Å, 2.0 Å	monomer	[66]
*Sphingomonas paucimobilis* UT26	1,3,4,6-tetrachloro-1,4-cyclohexadiene hydrolase linB complexed with 1,2-dichloropropane	*LinB*	1G42	Asp-108, Glu-132, and His-272	Asn-38 and Trp-109	1.8	monomer	[189]
*Burkholderia* Sp. Strain FA1	fluoroacetate dehalogenase	*FAcD*	1Y37	Asp-104, His-271, Asp-128	Trp-150 and His-149	1.5	homodimer	[191]
*Pseudomonas* Sp. Strain CBS-3	4-chlorobenzoyl-coenzyme A dehalogenase	*CoA*	1NZY	Asp-145 and His-90, Gly-114/Ala-121	Asp-145	1.8	hexamer	[166]

## References

[1] Negri A., Marco E., Damborsky J., Gago F. (2007). Stepwise dissection and visualization of the catalytic mechanism of haloalkane dehalogenase LinB using molecular dynamics simulations and computer graphics. J. Mol. Graph. Model..

[2] Chaudhry R.G., Chapalamadugu S. (1991). Biodegradation of halogenated organic compounds. Microbiol. Rev..

[3] Belkin S. (1992). Biodegradation of haloalkanes. Biodegradation.

[4] DePierre J.W., Nielson A.H. (2003). Mammalian toxicity of organic compounds of bromine and iodine. The Handbook of Environmental Chemistry.

[5] Bidmanova S., Chaloupkova R., Damborsky J., Prokop Z. (2010). Development of an enzymatic fibre-optic biosensor for detection of halogenated hydrocarbons. Anal. Bioanal. Chem..

[6] Vatsal A.A., Zinjarde S.S., RaviKumar A. (2017). Phenol Is the Initial Product Formed during Growth and Degradation of Bromobenzene by Tropical Marine Yeast, *Yarrowia lipolytica* NCIM 3589 via an Early Dehalogenation Step. Front. Microbiol..

[7] Puzyn T., Haranczyk M., Suzuki N., Sakurai T. (2011). Estimating persistence of brominated and chlorinated organic pollutants in the air, water, soil, and sediments with the QSPR-based classsification scheme. Mol. Divers..

[8] Wischnak C., Loffler F.E., Li J., Urbance J.W., Muller R. (1998). *Pseudomonas* sp. Strain 273, an Aerobic α,ω-Dichloroalkane-Degrading Bacterium. Appl. Environ. Microbiol..

[9] Copley S.D. (1997). Diverse mechanistic approaches to difficult chemical transformations: Microbial dehalogenation of chlorinated aromatic compounds. Chem. Biol..

[10] De Jong R.M., Dijkstra B.W. (2003). Structure and mechanism of bacterial dehalogenases: Different ways to cleave a carbon-halogen bond. Curr. Opin. Struct. Biol..

[11] Kurihara T., Esaki N. (2008). Bacterial hydrolytic dehalogenases and related enzymes: Occurrences, reaction mechanisms, and applications. Chem. Rec..

[12] Oakley A.J., Klvana M., Otyepka M., Nagata Y., Wilce M.C.J., Damborský J. (2004). Crystal structure of haloalkane dehalogenase LinB from *Sphingomonas paucimobilis* UT26 at 0.95 A resolution: Dynamics of catalytic residues. Biochemistry.

[13] Heeb N.V., Zindel D., Geueke B., Kohler H.P.E., Lienemann P. (2012). Biotransformation of hexabromocyclododecanes (HBCDs) with LinB-An HCH-converting bacterial enzyme. Environ. Sci. Technol..

[14] Koudelakova T., Bidmanova S., Dvorak P., Pavelka A., Chaloupkova R., Prokop Z., Damborský J. (2013). Haloalkane dehalogenases: Biotechnological applications. Biotechnol. J..

[15] Janssen D.B., Dinkla I.J.T., Poelarends G.J., Terpstra P. (2005). Bacterial degradation of xenobiotic compounds: Evolution and distribution of novel enzyme activities. Environ. Microbiol..

[16] United States Environmental Protection Agency (USEPA) (2009). Toxicological Review of Bromobenzene.

[17] Nikel P.I., Perez-Pantoja D., de Lorenzo V. (2013). Why are chlorinated pollutants so difficult to degrade aerobically? Redox stress limits 1,3-dichloprop-1-ene metabolism by *Pseudomonas pavonaceae*. Philos. Trans. R. Soc. B Biol. Sci..

[18] Franken S.M., Rozeboom H.J., Kalk K.H., Dijkstra B.W. (1991). Crystal structure of haloalkane dehalogenase: An enzyme to detoxify halogenated alkanes. EMBO J..

[19] Verschueren K.H.G., Seljée F., Rozeboom H.J., Kalk K.H., Dijkstra B.W. (1993). Crystallographic analysis of the catalytic mechanism of haloalkane dehalogenase. Nature.

[20] Prokop Z., Monincová M., Chaloupková R., Klvana M., Nagata Y., Janssen D.B., Damborský J. (2003). Catalytic mechanism of the maloalkane dehalogenase LinB from *Sphingomonas paucimobilis* UT26. J. Biol. Chem..

[21] Gehret J.J., Gu L., Geders T.W., Brown W.C., Gerwick L., Gerwick W.H., Sherman D.H., Smith J.L. (2012). Structure and activity of DmmA, a marine haloalkane dehalogenase. Protein Sci..

[22] Ridder I.S., Rozeboom J., Kalk K.H., Dijkstra B.W. (1999). Crystal Structures of Intermediates in the Dehalogenation of Haloalkanoates by L -2-Haloacid Dehalogenase. J. Biol. Chem..

[23] Hisano T., Hata Y., Fujii T., Liu J., Kurihara T., Esaki N., Soda K. (1996). Crystal Structure of l-2-Haloacid Dehalogenase from *Pseudomonas* sp. YL An α/β hydrolase structure that is different from the α/β hydrolase fold. J. Biol. Chem..

[24] Chovancová E., Kosinski J., Bujnicki J.M., Damborský J. (2007). Phylogenetic analysis of haloalkane dehalogenases. Proteins Struct. Funct. Bioinform..

[25] Kennes C., Pries F., Krooshof G.H., Bokma E., Kingma J., Janssen D.B. (1995). Replacement of tryptophan residues in haloalkane dehalogenase reduces halide binding and catalytic activity. Eur. J. Biochem..

[26] Krooshof G.H., Floris R., Tepper A.W., Janssen D.B. (1999). Thermodynamic analysis of halide binding to haloalkane dehalogenase suggests the occurrence of large conformational changes. Protein Sci..

[27] Tang X., Zhang R., Li Y., Zhang Q., Wang W. (2017). Enantioselectivity of haloalkane dehalogenase LinB on the degradation of 1,2-dichloropropane: A QM/MM study. Bioorg. Chem..

[28] Fetzner S., Lingens F. (1994). Bacterial dehalogenases: Biochemistry, genetics, and biotechnological applications. Microbiol. Mol. Biol. Rev..

[29] Fetzner S. (1998). Bacterial dehalogenation. Appl. Microbiol. Biotechnol..

[30] Smidt H., de Vos W.M. (2004). Anaerobic Microbial Dehalogenation. Annu. Rev. Microbiol..

[31] Leys D., Adrian L., Smidt H. (2013). Organohalide respiration: Microbes breathing chlorinated molecules. Philos. Trans. R. Soc. B Biol. Sci..

[32] Nijenhuis I., Kuntze K. (2016). Anaerobic microbial dehalogenation of organohalides-state of the art and remediation strategies. Curr. Opin. Biotechnol..

[33] Mészáros É., Imfeld G., Nikolausz M., Nijenhuis I. (2013). Occurrence of *dehalococcoides* and reductive dehalogenase genes in microcosms, a constructed wetland and groundwater from a chlorinated ethene contaminated field site as indicators for in situ reductive dehalogenation. Water Air Soil Pollut..

[34] Marzorati M., Balloi A., De Ferra F., Corallo L., Carpani G., Wittebolle L., Verstraete W., Daffonchio D. (2010). Bacterial diversity and reductive dehalogenase redundancy in a 1,2-dichloroethane-degrading bacterial consortium enriched from a contaminated aquifer. Microb. Cell Fact..

[35] Neumann A., Wohlfarth G., Diekert G. (1996). Purification and Characterization of Tetrachloroethene Reductive Dehalogenase from Dehalospirillum multivorans. J. Biol. Chem..

[36] Neumann A., Wohlfarth G., Diekert G. (1995). Properties of tetrachloroethene and trichloroethene dehalogenase of *Dehalospirillum multivorans*. Arch. Microbiol..

[37] Krasotkina J., Walters T., Maruya K.A., Ragsdale S.W. (2001). Characterization of the B12- and Iron-Sulfur-containing Reductive Dehalogenase from *Desulfitobacterium chlororespirans*. J. Biol. Chem..

[38] Van De Pas B.A., Smidt H., Hagen W.R., van der Oost J., Schraa G., Stams A.J.M., de Vos W.M. (1999). Purification and Molecular Characterization of ortho -Chlorophenol Reductive Dehalogenase, a Key Enzyme of Halorespiration in *Desulfitobacterium dehalogenans*. Biochemistry.

[39] Maillard J., Schumacher W., Vazquez F., Regeard C., Hagen W.R., Holliger C. (2003). Characterization of the corrinoid iron-sulfur protein tetrachloroethene reductive dehalogenase of *Dehalobacter restrictus*. Appl. Environ. Microbiol..

[40] Loffler F.E., Sanford R.A., Tiedje J.M. (1996). Initial characterization of a reductive dehalogenase from *Desulfitobacterium chlororespirans* Co23. Appl. Environ. Microbiol..

[41] Ni S., Fredrickson J.K., Acteriol J.B. (1995). Purification and characterization of a novel 3-chlorobenzoate- reductive dehalogenase from the cytoplasmic membrane of *Desulfomonile tiedjei* DCB-1. J. Bacteriol..

[42] DeWeerd K.A., Suflita J.M. (1990). Anaerobic aryl reductive dehalogenation of halobenzoates by cell extracts of “*Desulfomonile tiedjei*”. Appl. Environ. Microbiol..

[43] Mohn W.W., Tiedje J.M. (1992). Microbial reductive dehalogenation. Microbiol. Rev..

[44] Payne K.A.P., Quezada C.P., Fisher K., Dunstan M.S., Collins F.A., Sjuts H., Levy C., Hay S., Rigby S.E.J., Leys D. (2014). Reductive dehalogenase structure suggests a mechanism for B12-dependent dehalogenation. Nature.

[45] Banerjee R., Ragsdale S.W. (2003). The Many Faces of Vitamin B_12_: Catalysis by Cobalamin-Dependent Enzymes. Annu. Rev. Biochem..

[46] Poelarends G.J., Veetil V.P., Whitman C.P. (2008). The chemical versatility of the β–α–β fold: Catalytic promiscuity and divergent evolution in the tautomerase superfamily. Cell. Mol. Life Sci..

[47] Tsang J.S.H., Sallis P.J., Bull A.T., Hardman D.J. (1988). A monobromoacetate dehalogenase from *Pseudomonas* cepacia MBA4. Arch. Microbiol..

[48] Schwarze R., Brokamp A., Schmidt F. (1997). Isolation and Characterization of Dehalogenases from 2,2-Dichloropropionate-Degrading Soil Bacteria. Curr. Microbiol..

[49] Liu J.Q., Kurihara T., Miyagi M., Esaki N., Soda K. (1995). Reaction mechanism of L-2-haloacid dehalogenase of *Pseudomonas* sp. YL: Identification of Asp10 as the active site nucleophile by ^18^O incorporation experiments. J. Biol. Chem..

[50] Omi R., Jitsumori K., Yamauchi T., Ichiyama S., Kurihara T., Esaki N., Kamiya N., Hirotsu K., Miyahara I. (2007). Expression, purification and preliminary X-ray characterization of DL-2-haloacid dehalogenase from Methylobacterium sp. CPA1. Acta Crystallogr. Sect. F Struct. Biol. Cryst. Commun..

[51] Nardi-Dei V., Kurihara T., Park C., Miyagi M., Tsunasawa S., Soda K., Esaki N. (1999). dl-2-haloacid dehalogenase from *Pseudomonas* sp. 113 is a new class of dehalogenase catalyzing hydrolytic dehalogenation not involving enzyme- substrate ester intermediate. J. Biol. Chem..

[52] Arai R., Kukimoto-niino M., Kuroishi C., Bessho Y., Shirouzu M. (2006). Crystal structure of the probable haloacid dehalogenase PH0459 from *Pyrococcus horikoshii* OT3. Protein Sci..

[53] Ridder I.S., Rozeboom H.J., Kalk K.H., Janssen D.B., Dijkstra B.W. (1997). Three-dimensional structure of l-2-haloacid dehalogenase from *Xanthobacter autotrophicus* GJ10 complexed with the substrate-analogue formate. J. Biol. Chem..

[54] Schmidberger J.W., Wilce J.A., Tsang J.S.H., Wilce M.C.J. (2007). Crystal Structures of the Substrate Free-enzyme, and Reaction Intermediate of the HAD Superfamily Member, Haloacid Dehalogenase DehIVa from Burkholderia cepacia MBA4. J. Mol. Biol..

[55] Asmara W., Murdiyatmo U., Baines A.J., Bull A.T., Hardman D.J. (1993). Protein engineering of the 2-haloacid halidohydrolase IVa from *Pseudomonas* cepacia MBA4. Biochem. J..

[56] Pang B.C.M., Tsang J.S.H. (2001). Mutagenic analysis of the conserved residues in dehalogenase IVa of *Burkholderia cepacia* MBA4. FEMS Microbiol. Lett..

[57] Schneider B., Müller R., Frank R., Lingens F. (1993). Site-Directed Mutagenesis of the 2-Haloalkanoic Acid Dehalogenase I Gene from *Pseudomonas* sp. Strain CBS3 and its Effect on Catalytic Activity. Biol. Chem. Hoppe Seyler.

[58] Tsang J.S.H., Pang B.C.M. (2000). Identification of the Dimerization Domain of Dehalogenase IVa of *Burkholderia cepacia* MBA4. Appl. Environ. Microbiol..

[59] Kuznetsova E., Nocek B., Brown G., Makarova K.S., Flick R., Wolf Y.I., Khusnutdinova A., Evdokimova E., Jin K., Tan K. (2015). Functional Diversity of Haloacid Dehalogenase Superfamily Phosphatases from *Saccharomyces cerevisiae*: Biochemical, Structural, and Evolutionary Insights. J. Biol. Chem..

[60] Furukawa K. (2000). Biochemical and genetic bases of microbial degradation of polychlorinated biphenyls (PCBs). J. Gen. Appl. Microbiol..

[61] Janssen D.B. (2004). Evolving haloalkane dehalogenases. Curr. Opin. Chem. Biol..

[62] Koudelakova T., Chovancova E., Brezovsky J., Monincova M., Fortova A., Jarkovsky J., Damborsky J. (2011). Substrate specificity of haloalkane dehalogenases. Biochem. J..

[63] Palau J., Cretnik S., Shouakar-Stash O., Höche M., Elsner M., Hunkeler D. (2014). C and Cl Isotope Fractionation of 1,2-Dichloroethane Displays Unique δ^13^C/δ^37^Cl Patterns for Pathway Identification and Reveals Surprising C–Cl Bond Involvement in Microbial Oxidation. Environ. Sci. Technol..

[64] Pikkemaat M.G., Linssen A.B.M., Berendsen H.J.C., Janssen D.B. (2002). Molecular dynamics simulations as a tool for improving protein stability. Protein Eng. Des. Sel..

[65] Newman J., Peat T.S., Richard R., Kan L., Swanson P.E., Affholter J.A., Holmes I.H., Schindler J.F., Unkefer C.J., Terwilliger T.C. (1999). Haloalkane Dehalogenases: Structure of a Rhodococcus Enzyme. Biochemistry.

[66] Marek J., Vevodova J., Smatanova I.K., Nagata Y., Svensson L.A., Newman J., Takagi M., Damborsky J. (2000). Crystal structure of the haloalkane dehalogenase from *Sphingomonas paucimobilis* UT26. Biochemistry.

[67] Kutý M., Damborský J., Prokop M., Koča J. (1998). A Molecular Modeling Study of the Catalytic Mechanism of Haloalkane Dehalogenase. 2. Quantum Chemical Study of Complete Reaction Mechanism. J. Chem. Inf. Comput. Sci..

[68] Hladilkova J., Prokop Z., Chaloupkova R., Damborsky J., Jungwirth P. (2013). Release of halide ions from the buried active site of the haloalkane dehalogenase LinB revealed by stopped-flow fluorescence analysis and free energy calculations. J. Phys. Chem. B.

[69] Pavlová M., Klvaňa M., Jesenská A., Prokop Z., Konečná H., Sato T., Tsuda M., Nagata Y., Damborský J. (2007). The identification of catalytic pentad in the haloalkane dehalogenase DhmA from *Mycobacterium avium* N85: Reaction mechanism and molecular evolution. J. Struct. Biol..

[70] Buryska T., Daniel L., Kunka A., Brezovsky J., Damborsky J., Prokop Z. (2016). Discovery of Novel Haloalkane Dehalogenase Inhibitors. Appl. Environ. Microbiol..

[71] Chaloupková R., Sýkorová J., Prokop Z., Jesenská A., Monincová M., Pavlová M., Tsuda M., Nagata Y., Damborský J. (2003). Modification of Activity and Specificity of Haloalkane Dehalogenase from *Sphingomonas paucimobilis* UT26 by Engineering of Its Entrance Tunnel. J. Biol. Chem..

[72] Chan P.W.Y., Yakunin A.F., Edwards E.A., Pai E.F. (2011). Mapping the Reaction Coordinates of Enzymatic Defluorination. J. Am. Chem. Soc..

[73] Au K.G., Walsh C.T. (1984). Stereochemical studies on a plasmid-coded fluoroacetate halidohydrolase. Bioorg. Chem..

[74] Kurihara T., Yamauchi T., Ichiyama S., Takahata H., Esaki N. (2003). Purification, characterization, and gene cloning of a novel fluoroacetate dehalogenase from *Burkholderia* sp. FA1. J. Mol. Catal. B Enzym..

[75] Goldman P. (1969). The carbon-fluorine bond in compounds of biological interest. Science.

[76] Holmquist M. (2000). Alpha Beta-Hydrolase Fold Enzymes Structures, Functions and Mechanisms. Curr. Protein Pept. Sci..

[77] Kamachi T., Nakayama T., Shitamichi O., Jitsumori K., Kurihara T., Esaki N., Yoshizawa K. (2009). The catalytic mechanism of fluoroacetate dehalogenase: A computational exploration of biological dehalogenation. Chem. A Eur. J..

[78] Li Y., Zhang R., Du L., Zhang Q., Wang W. (2016). Catalytic mechanism of C–F bond cleavage: Insights from QM/MM analysis of fluoroacetate dehalogenase. Catal. Sci. Technol..

[79] Kawasaki H., Tsuda K., Matsushita I., Tonomura K. (1992). Lack of homology between two haloacetate dehalogenase genes encoded on a plasmid from *Moraxella* sp. strain B. J. Gen. Microbiol..

[80] Leong L.E.X., Khan S., Davis C.K., Denman S.E., McSweeney C.S. (2017). Fluoroacetate in plants—A review of its distribution, toxicity to livestock and microbial detoxification. J. Anim. Sci. Biotechnol..

[81] Liu J.Q., Kurihara T., Ichiyama S., Miyagi M., Tsunasawa S., Kawasaki H., Soda K., Esaki N. (1998). Reaction mechanism of fluoroacetate dehalogenase from *Moraxella* sp. B. J. Biol. Chem..

[82] Chan W.Y., Wong M., Guthrie J., Savchenko A.V., Yakunin A.F., Pai E.F., Edwards E.A. (2010). Sequence- and activity-based screening of microbial genomes for novel dehalogenases. Microb. Biotechnol..

[83] Luo L., Taylor K.L., Xiang H., Wei Y., Zhang W., Dunaway-Mariano D. (2001). Role of active site binding interactions in 4-chlorobenzoyl-coenzyme A dehalogenase catalysis. Biochemistry.

[84] Yang G., Liang P.-H., Dunaway-Mariano D. (1994). Evidence for Nucleophilic Catalysis in the Aromatic Substitution Reaction Catalyzed by (4-Chlorobenzoyl) coenzyme A Dehalogenase. Biochemistry.

[85] Crooks G.P., Xu L., Barkley R.M., Copley S.D. (1995). Exploration of Possible Mechanisms for 4-Chlorobenzoyl CoA Dehalogenase: Evidence for an Aryl—Enzyme Intermediate. J. Am. Chem. Soc..

[86] Krooshof G.H., Kwant E.M., Damborský J., Koča J., Janssen D.B. (1997). Repositioning the Catalytic Triad Aspartic Acid of Haloalkane Dehalogenase: Effects on Stability, Kinetics, and Structure. Biochemistry.

[87] Smilda T., Kamminga A.H., Reinders P., Baron W., van Hylckama Vlieg J.E.T., Beintema J.J. (2001). Enzymic and Structural Studies on Drosophila Alcohol Dehydrogenase and Other Short-Chain Dehydrogenases/Reductases. J. Mol. Evol..

[88] Kulakov L.A., Poelarends G.J., Janssen D.B., Larkin M.J. (1999). Characterization of IS2112, a new insertion sequence from *Rhodococcus*, and its relationship with mobile elements belonging to the IS110 family. Microbiology.

[89] Scholtz R., Messi F., Leisinger T., Cook A.M. (1988). Three dehalogenases and physiological restraints in the biodegradation of haloalkanes by *Arthrobacter* sp. strain HA1. Appl. Environ. Microbiol..

[90] Casellas M., Grifoll M., Bayona J.M., Solanas A.M. (1997). New metabolites in the degradation of fluorene by *Arthrobacter* sp. strain F101. Appl. Environ. Microbiol..

[91] Van den Wijngaard A.J., Reuvekamp P.T., Janssen D.B. (1991). Purification and Characterization of Haloalcohol Dehalogenase. J. Bacteriol..

[92] Yao Y., Tang H., Su F., Xu P. (2015). Comparative genome analysis reveals the molecular basis of nicotine degradation and survival capacities of Arthrobacter. Sci. Rep..

[93] Husserl J., Hughes J.B., Spain J.C. (2012). Key enzymes enabling the growth of *Arthrobacter* sp. Strain JBH1 with nitroglycerin as the sole source of carbon and nitrogen. Appl. Environ. Microbiol..

[94] Jain R.K., Dreisbach J.H., Spain J.C. (1994). Biodegradation of *p*-Nitrophenol via 1,2,4-Benzenetriol by an *Arthrobacter* sp.. Appl. Environ. Microbiol..

[95] Kawasaki H., Yahara H., Tonomura K. (1981). Isolation and Characterization of Plasmid pUOl Mediating Dehalogenation of Haloacetate and Mercury Resistance in *Moraxella* sp. B. Agric. Biol. Chem..

[96] Kawasaki H., Toyama T., Maeda T., Nishino H., Tonomura K. (1994). Cloning and sequence analysis of a plasmid-encoded 2-haloacid dehalogenase gene from *Pseudomonas putida* no. 109. Biosci. Biotechnol. Biochem..

[97] Van der Ploeg J., Van Hall G., Janssen D.B. (1991). Characterization of the haloacid dehalogenase from *Xanthobacter autotrophicus* GJ10 and sequencing of the dhlB gene. J. Bacteriol..

[98] Jones D.H., Barth P.T., Byrom D., Thomas C.M. (1992). Nucleotide sequence of the structural gene encoding a 2-haloalkanoic acid dehalogenase of *Pseudomonas putida* strain AJ1 and purification of the encoded protein. J. Gen. Microbiol..

[99] Schmidberger J.W., Wilce J.A., Weightman A.J., Wilce M.C.J. (2008). Purification, crystallization and preliminary crystallographic analysis of DehI, a group I α-haloacid dehalogenase from *Pseudomonas putida* strain PP3. Acta Crystallogr. Sect. F Struct. Biol. Cryst. Commun..

[100] Holloway P., Knoke K.L., Trevors J.T., Lee H. (1998). Alteration of the substrate range of haloalkane dehalogenase by site-directed mutagenesis. Biotechnol. Bioeng..

[101] Kohler R., Brokamp A., Schwarze R., Reiting R.H., Schmidt F.R.J. (1998). Characteristics and DNA-sequence of a cryptic haloalkanoic acid dehalogenase from *Agrobacterium tumefaciens* RS5. Curr. Microbiol..

[102] De Jong R.M., Brugman W., Poelarends G.J., Whitman C.P., Dijkstra B.W. (2004). The X-ray Structure of trans-3-Chloroacrylic Acid Dehalogenase Reveals a Novel Hydration Mechanism in the Tautomerase Superfamily. J. Biol. Chem..

[103] Kurata A., Kurihara T., Kamachi H., Esaki N. (2004). Asymmetric reduction of 2-chloroacrylic acid to (*S*)-2-chloropropionic acid by a novel reductase from *Burkholderia* sp. WS. Tetrahedron Asymmetry.

[104] Li Y.F., Hata Y., Fujii T., Hisano T., Nishihara M., Kurihara T., Esaki N. (1998). Crystal structures of reaction intermediates of L-2-haloacid dehalogenase and implications for the reaction mechanism. J. Biol. Chem..

[105] Müller R., Thiele J., Klages U., Lingens F. (1984). Incorporation of [18O]water into 4-hydroxybenzoic acid in the reaction of 4-chlorobenzoate dehalogenase from *Pseudomonas* sp. CBS 3. Biochem. Biophys. Res. Commun..

[106] Liu J., Kurihara T., Esaki N., Soda K. (1994). Reconsideration Acid of the Essential Dehalogenase Role of a Histidine Residue of L-2-Halo Acid Dehalogenase Role. J. Biochem..

[107] Rye C.A., Isupov M.N., Lebedev A.A., Littlechild J.A. (2009). Biochemical and structural studies of a l-haloacid dehalogenase from the thermophilic archaeon *Sulfolobus tokodaii*. Extremophiles.

[108] Novak H.R., Sayer C., Isupov M.N., Paszkiewicz K., Gotz D., Mearns Spragg A., Littlechild J.A. (2013). Marine Rhodobacteraceae l-haloacid dehalogenase contains a novel His/Glu dyad that could activate the catalytic water. FEBS J..

[109] Egland P.G., Gibson J., Harwood C.S. (2001). Reductive, Coenzyme A-Mediated Pathway for 3-Chlorobenzoate Degradation in the Phototrophic Bacterium *Rhodopseudomonas palustris*. Appl. Environ. Microbiol..

[110] Tratsiak K., Degtjarik O., Drienovska I., Chrast L., Rezacova P., Kuty M., Chaloupkova R., Damborsky J., Kuta Smatanova I. (2013). Crystallographic analysis of new psychrophilic haloalkane dehalogenases: DpcA from *Psychrobacter cryohalolentis* K5 and DmxA from *Marinobacter* sp. ELB17. Acta Crystallogr. Sect. F Struct. Biol. Cryst. Commun..

[111] Drienovska I., Chovancova E., Koudelakova T., Damborsky J., Chaloupkova R. (2012). Biochemical characterization of a novel haloalkane dehalogenase from a cold-adapted bacterium. Appl. Environ. Microbiol..

[112] Li A., Shao Z. (2014). Biochemical characterization of a haloalkane dehalogenase DadB from *Alcanivorax dieselolei* B-5. PLoS ONE.

[113] Senoo K., Wada H. (1989). Isolation and identification of an aerobic γ-HCH-decomposing bacterium from soil. Soil Sci. Plant Nutr..

[114] Wada H., Senoo K., Takai Y. (1989). Rapid degradation of γ-HCH in upland soil after multiple applications. Soil Sci. Plant Nutr..

[115] Zhou L., Marks T.S., Poh R.P.C., Smith R.J., Chowdhry B.Z., Smith A.R.W. (2004). The purification and characterisation of 4-chlorobenzoate:CoA ligase and 4-chlorobenzoyl CoA dehalogenase from *Arthrobacter* sp. strain TM-1. Biodegradation.

[116] Bagherbaigi S., Gicana R.G., Lamis R.J., Nemati M., Huyop F. (2013). Characterisation of *Arthrobacter* sp. S1 that can degrade α and β-haloalkanoic acids isolated from contaminated soil. Ann. Microbiol..

[117] Mazumdar P.A., Hulecki J.C., Cherney M.M., Garen C.R., James M.N.G. (2008). X-ray crystal structure of *Mycobacterium tuberculosis* haloalkane dehalogenase Rv2579. Biochim. Biophys. Acta Proteins Proteom..

[118] Ichiyama S., Kurihara T., Miyagi M., Galkin A., Tsunasawa S., Kawasaki H., Esaki N. (2002). Catalysis-linked inactivation of fluoroacetate dehalogenase by ammonia: A novel approach to probe the active-site environment. J. Biochem..

[119] Stringfellow J.M., Cairns S.S., Cornish A., Cooper R.A. (1997). Haloalkanoate dehalogenase II (DehE) of a *Rhizobium* sp. molecular analysis of the gene and formation of carbon monoxide from trihaloacetate by the enzyme. Eur. J. Biochem..

[120] Prokop Z., Sato Y., Brezovsky J., Mozga T., Chaloupkova R., Koudelakova T., Jerabek P., Stepankova V., Natsume R., Van Leeuwen J.G.E. (2010). Enantioselectivity of haloalkane dehalogenases and its modulation by surface loop engineering. Angew. Chem. Int. Ed..

[121] Okai M., Ohtsuka J., Imai L.F., Mase T., Moriuchi R., Tsuda M., Nagata K., Nagata Y., Tanokura M. (2013). Crystal structure and site-directed mutagenesis analyses of haloalkane dehalogenase linB from *Sphingobium* sp. Strain MI1205. J. Bacteriol..

[122] Magnuson J.K., Romine M.F., Burris D.R., Kingsley M.T. (2000). Trichloroethene reductive dehalogenase from *Dehalococcoides ethenogenes*: Sequence of tceA and substrate range characterization. Appl. Environ. Microbiol..

[123] Krajmalnik-Brown R., Holscher T., Thomson I.N., Saunders F.M., Ritalahti K.M., Loffler F.E. (2004). Genetic Identification of a Putative Vinyl Chloride Reductase in strain BAV1. Appl. Environ. Microbiol..

[124] Guan L., Yabuki H., Okai M., Ohtsuka J., Tanokura M. (2014). Crystal structure of the novel haloalkane dehalogenase DatA from *Agrobacterium tumefaciens* C58 reveals a special halide-stabilizing pair and enantioselectivity mechanism. Appl. Microbiol. Biotechnol..

[125] Prudnikova T., Mozga T., Rezacova P., Chaloupkova R., Sato Y., Nagata Y., Brynda J., Kuty M., Damborsky J., Kuta Smatanova I. (2009). Crystallization and preliminary X-ray analysis of a novel haloalkane dehalogenase DbeA from *Bradyrhizobium elkani* USDA94. Acta Crystallogr. Sect. F Struct. Biol. Cryst. Commun..

[126] Lohner S.T., Spormann A.M. (2013). Identification of a reductive tetrachloroethene dehalogenase in *Shewanella sediminis*. Philos. Trans. R. Soc. Lond. B Biol. Sci..

[127] De Jong R.M. (2003). Structure and mechanism of a bacterial haloalcohol dehalogenase: A new variation of the short-chain dehydrogenase/reductase fold without an NAD(P)H binding site. EMBO J..

[128] Benedetti I., de Lorenzo V., Nikel P.I. (2016). Genetic programming of catalytic *Pseudomonas putida* biofilms for boosting biodegradation of haloalkanes. Metab. Eng..

[129] Yokota T., Fuse H., Omori T., Minoda Y. (1986). Microbial Dehalogenation of Haloalkanes Mediated by Oxygenase or Halidohydrolase. Agric. Biol. Chem..

[130] Jesenská A., Sedláček I., Damborský J. (2000). Dehalogenation of haloalkanes by *Mycobacterium tuberculosis* H37Rv and other mycobacteria. Appl. Environ. Microbiol..

[131] Poelarends G.J., Van Hylckama Vlieg J.E.T., Marchesi J.R., Dos Santos L.M.F., Janssen D.B. (1999). Degradation of 1,2-dibromoethane by *Mycobacterium* sp. strain GP1. J. Bacteriol..

[132] Kulakova A.N., Larkin M.J., Kulakov L.A. (1997). The plasmid-located haloalkane dehalogenase gene from *Rhodococcus rhodochrous* NCIMB 13064. Microbiology.

[133] Kmunicek J., Hynkova K., Jedlicka T., Nagata Y., Negri A., Gago F., Wade R.C., Damborsky J. (2005). Quantitative Analysis of Substrate Specificity of Haloalkane Dehalogenase LinB from *Sphingomonas paucimobilis* UT26. Biochemistry.

[134] Raina V., Hauser A., Buser H.R., Rentsch D., Sharma P., Lal R., Holliger C., Poiger T., Muller M.D., Kohler H.P.E. (2007). Hydroxylated metabolites of β- and δ-hexachlorocyclohexane: Bacterial formation, stereochemical configuration, and occurrence in groundwater at a former production site. Environ. Sci. Technol..

[135] Bala K., Geueke B., Miska M.E., Rentsch D., Poiger T., Dadhwal M., Lal R., Holliger C., Kohler H.P.E. (2012). Enzymatic conversion of ε-hexachlorocyclohexane and a heptachlorocyclohexane isomer, two neglected components of technical hexachlorocyclohexane. Environ. Sci. Technol..

[136] Nagata Y., Miyauchi K., Damborsky J., Manova K., Ansorgova A., Takagi M. (1997). Purification and characterization of a haloalkane dehalogenase of a new substrate class from a γ-hexachlorocyclohexane—Degrading bacterium, *Sphingomonas paucimobilis* UT26. Appl. Environ. Microbiol..

[137] Mowafy A.M., Kurihara T., Kurata A., Uemura T., Esaki N. (2010). 2-Haloacrylate Hydratase, a New Class of Flavoenzyme That Catalyzes the Addition of Water To the Substrate for Dehalogenation. Appl. Environ. Microbiol..

[138] Horvat C.M., Wolfenden R.V. (2005). A persistent pesticide residue and the unusual catalytic proficiency of a dehalogenating enzyme. Proc. Natl. Acad. Sci. USA.

[139] Kurata A., Kurihara T., Kamachi H., Esaki N. (2005). 2-Haloacrylate reductase, a novel enzyme of the medium chain dehydrogenase/reductase superfamily that catalyzes the reduction of a carbon-carbon double bond of unsaturated organohalogen compounds. J. Biol. Chem..

[140] Quamrul Hasan A.K.M., Takada H., Koshikawa H., Liu J.Q., Kurihara T., Esaki N., Soda K. (1994). Two Kinds of 2-Halo Acid Dehalogenases from *Pseudomonas* sp. YL Induced by 2-Chloroacrylate and 2-Chloropropionate. Biosci. Biotechnol. Biochem..

[141] Tsang J.S.H., Sam L. (1999). Cloning and characterization of a cryptic haloacid dehalogenase from *Burkholderia cepacia* MBA4. J. Bacteriol..

[142] Park C., Kurihara T., Yoshimura T., Soda K., Esaki N. (2003). A new DL-2-haloacid dehalogenase acting on 2-haloacid amides: Purification, characterization, and mechanism. J. Mol. Catal. B Enzym..

[143] Motosugi K., Esaki N., Soda K. (1982). Purification and properties of a new enzyme, DL-2-haloacid dehalogenase, from *Pseudomonas* sp.. J. Bacteriol..

[144] Huyop F., Sudi I.Y. (2012). D-specific dehalogenases, a review. Biotechnol. Biotechnol. Equip..

[145] Weightman A.J., Weightman A.L., Slater J.H. (1982). Stereospecificity of 2-Monochloropropionate Dehalogenation by the Two Dehalogenases of *Pseudomonas putida* PP3: Evidence for Two Different Dehalogenation Mechanisms. Microbiology.

[146] O’Hagan D., Schaffrath C., Cobb S.L., Hamilton J.T.G., Murphy C.D. (2002). Biochemistry: Biosynthesis of an organofluorine molecule. Nature.

[147] Van Den Berg M.A., Steensma H.Y. (1997). Expression cassettes for formaldehyde and fluoroacetate resistance, two dominant markers in *Saccharomyces cerevisiae*. Yeast.

[148] Tamura T., Wada M., Esaki N., Soda K. (1995). Synthesis of fluoroacetate from fluoride, glycerol, and beta- hydroxypyruvate by Streptomyces cattleya. J. Bacteriol..

[149] Chen H., Wang H., Wang T., Huang S., Zang X., Li S., Jiang J. (2016). Identification of the metal center of chlorothalonil hydrolytic dehalogenase and enhancement of catalytic efficiency by directed evolution. Appl. Environ. Biotechnol..

[150] Taş N., Van Eekert M.H.A., De Vos W.M., Smidt H. (2010). The little bacteria that can - Diversity, genomics and ecophysiology of “*Dehalococcoides*” spp. in contaminated environments. Microb. Biotechnol..

[151] Nelson J.L., Jiang J., Zinder S.H. (2014). Dehalogenation of chlorobenzenes, dichlorotoluenes, and tetrachloroethene by three *Dehalobacter* spp.. Environ. Sci. Technol..

[152] Manchester M.J., Hug L.A., Zarek M., Zila A., Edwards E.A. (2012). Discovery of a trans-dichloroethene-respiring *Dehalogenimonas* species in the 1,1,2,2-tetrachloroethane-dechlorinating WBC-2 consortium. Appl. Environ. Microbiol..

[153] Zhao S., Ding C., He J. (2015). Detoxification of 1,1,2-trichloroethane to ethene by desulfitobacterium and identification of its functional reductase gene. PLoS ONE.

[154] Low A., Shen Z., Cheng D., Rogers M.J., Lee P.K.H., He J. (2015). A comparative genomics and reductive dehalogenase gene transcription study of two chloroethene-respiring bacteria, *Dehalococcoides mccartyi* strains MB and 11a. Sci. Rep..

[155] Janssen D.B., Oppentocht J.E., Poelarends G.J. (2001). Microbial dehalogenation. Curr. Opin. Biotechnol..

[156] Beil S., Timmis K.N., Pieper D.H. (1999). Genetic and biochemical analyses of the tec operon suggest a route for evolution of chlorobenzene degradation genes. J. Bacteriol..

[157] Anandarajah K., Kiefer P.M., Donohoe B.S., Copley S.D. (2000). Recruitment of a double bond isomerase to serve as a reductive dehalogenase during biodegradation of pentachlorophenol. Biochemistry.

[158] Armstrong R.N. (1997). Structure, Catalytic Mechanism, and Evolution of the Glutathione Transferases. Chem. Res. Toxicol..

[159] Ahn Y., Rhee S., Fennell D.E., Kerkhof J., Hentschel U., Häggblom M.M., Kerkhof L.J., Ha M.M. (2003). Reductive Dehalogenation of Brominated Phenolic Compounds by Microorganisms Associated with the Marine Sponge *Aplysina aerophoba*. Appl. Environ. Microb..

[160] Weigold P., El-Hadidi M., Ruecker A., Huson D.H., Scholten T., Jochmann M., Kappler A., Behrens S. (2016). A metagenomic-based survey of microbial (de)halogenation potential in a German forest soil. Sci. Rep..

[161] Butler A., Sandy M. (2009). Mechanistic considerations of halogenating enzymes. Nature.

[162] Hofrichter M., Ullrich R., Pecyna M.J., Liers C., Lundell T. (2010). New and classic families of secreted fungal heme peroxidases. Appl. Microbiol. Biotechnol..

[163] Hager P., Morris D.R., Brown S., Eberwein H. (1966). Chloroperoxidase. J. Biol. Chem..

[164] Wuosmaa A.M., Hager L.P. (1982). Methyl Chloride Transferase: A Carbocation Route for Biosynthesis of Halometabolites. Science.

[165] Ollis D.L., Cheah E., Cygler M., Dijkstra B., Frolow F., Franken S.M., Harel M., Remington S.J., Silman I., Schrag J. (1992). The alpha/beta hydrolase fold. Protein Eng..

[166] Benning M.M., Taylor K.L., Liu R.-Q., Yang G., Xiang H., Wesenberg G., Dunaway-Mariano D., Holden H.M. (1996). Structure of 4-Chlorobenzoyl Coenzyme A Dehalogenase Determined to 1.8 Å Resolution: An Enzyme Catalyst Generated via Adaptive Mutation. Biochemistry.

[167] Van Hylckama Vlieg J.E.T., Vlieg H., Tang L., Spelberg J.H.L., Smilda T.I.M., Poelarends G.J., Bosma T., van Merode A.E.J., Fraaije M.W., Janssen D.B. (2001). Halohydrin Dehalogenases Are Structurally and Mechanistically Related to Short-Chain Dehydrogenases/Reductases. J. Bacteriol..

[168] Russell R.B., Sternberg M.J.E. (1997). Two new examples of protein structural similarities within the structure—Function twilight zone. Protein Eng..

[169] Stammers D.K., Ren J., Leslie K., Nichols C.E., Lamb H.K., Cocklin S., Dodds A., Hawkins A.R. (2001). The structure of the negative transcriptional regulator NmrA reveals a structural superfamily which includes the short-chain dehydrogenase/reductases. EMBO J..

[170] Oppermann U., Filling C., Hult M., Shafqat N., Wu X., Lindh M., Shafqat J., Nordling E., Kallberg Y., Persson B. (2003). Short-chain dehydrogenases/reductases (SDR): The 2002 update. Chem. Biol. Interact..

[171] Filling C., Berndt K.D., Benach J., Knapp S., Prozorovski T., Nordling E., Ladenstein R., Jörnvall H., Oppermann U. (2002). Critical residues for structure and catalysis in short-chain dehydrogenases/reductases. J. Biol. Chem..

[172] Jomvall H., Persson B., Jörnvall H., Persson B., Krook M., Atrian S., Gonzàlez-Duarte R., Jeffery J., Ghosh D. (1995). Short-chain dehydrogenases/reductases(SDR). Biochemistry.

[173] Ridder I.S., Dijkstra B.W. (1999). Identification of the Mg2+-binding site in the P-type ATPase and phosphatase members of the HAD (haloacid dehalogenase) superfamily by structural similarity to the response regulator protein CheY. Biochem. J..

[174] Taylor K.L., Liu R.Q., Liang P.H., Price J., Dunaway-Mariano D., Tonge P.J., Clarkson J., Carey P.R. (1995). Evidence for Electrophilic Catalysis in the 4-Chlorobenzoyl-CoA Dehalogenase Reaction: UV, Raman, and 13C-NMR Spectral Studies of Dehalogenase Complexes of Benzoyl-CoA Adducts. Biochemistry.

[175] Peat T.S., Newman J., Balotra S., Lucent D., Warden A.C., Scott C. (2015). The structure of the hexameric atrazine chlorohydrolase AtzA. Acta Crystallogr. Sect. D Biol. Crystallogr..

[176] Seffernick J.L., Reynolds E., Fedorov A.A., Fedorov E., Almo S.C., Sadowsky M.J., Wackett L.P. (2010). X-ray structure and mutational analysis of the atrazine chlorohydrolase TrzN. J. Biol. Chem..

[177] Streltsov V.A., Prokop Z., Damborský J., Nagata Y., Oakley A., Wilce M.C.J. (2003). Haloalkane dehalogenase LinB from *Sphingomonas paucimobilis* UT26: X-ray crystallographic studies of dehalogenation of brominated substrates. Biochemistry.

[178] De Jong R.M., Bazzacco P., Poelarends G.J., Johnson W.H., Yoon J.K., Burks E.A., Serrano H., Thunnissen A.M.W.H., Whitman C.P., Dijkstra B.W. (2007). Crystal structures of native and inactivated *cis*-3-chloroacrylic acid dehalogenase: Structural basis for substrate specificity and inactivation by (R)-oxirane-2-carboxylate. J. Biol. Chem..

[179] Siwek A., Omi R., Hirotsu K., Jitsumori K., Esaki N., Kurihara T., Paneth P. (2013). Binding modes of DL-2-haloacid dehalogenase revealed by crystallography, modeling and isotope effects studies. Arch. Biochem. Biophys..

[180] Widersten M. (1998). Heterologous Expression in Escherichia coli of Soluble Active-Site Random Mutants of Haloalkane Dehalogenase from *Xanthobacter autotrophicu*s GJ10 by Coexpression of Molecular Chaperonins GroEL/ES 1. Protein Exp. Purif..

[181] Liu J., Kurihara T., Miyagi M., Tsunasawa S., Nishihara M., Esaki N., Soda K. (1997). Paracatalytic Inactivation of L-2-Haloacid Dehalogenase from *Pseudomonas* sp. YL by Hydroxylamine. Biochemistry.

[182] Pries F., Van Den Wijngaard A.J., Bos R., Pentenga M., Janssen D.B. (1994). The role of spontaneous cap domain mutations in haloalkane dehalogenase specificity and evolution. J. Biol. Chem..

[183] Pries F., Kingma J., Krooshof G.H., Jeronimus-Stratingh C.M., Bruins A.P., Janssen D.B. (1995). Histidine 289 is essential for hydrolysis of the alkyl-enzyme intermediate of haloalkane dehalogenase. J. Biol. Chem..

[184] Otyepka M., Banáš P., Magistrato A., Carloni P., Damborský J. (2007). Second step of hydrolytic dehalogenation in haloalkane dehalogenase investigated by QM/MM methods. Proteins Struct. Funct. Bioinform..

[185] Campbell D.W., Müller C., Reardon K.F. (2006). Development of a fibre-optic enzymatic biosensor for 1,2-dichloroethane. Biotechnol. Lett..

[186] Kurihara T., Liu J.-Q., Nardi-Dei V., Koshikawa H., Nobuyoshi E., Soda K. (1995). Comprehensive Site-Directed Mutagenesis of L-2-Halo Acid Dehalogenase to to Probe Catalytic Amino Residues. J. Biochem.

[187] Murdiyatmo U., Asmara W., Tsang J.S., Baines A.J., Bull A.T., Hardman D.J. (1992). Protein engineering of the 2-haloacid halidohydrolase IVa from *Pseudomonas cepacia* MBA4. Biochem. J..

[188] Ohana R.F., Encell L.P., Zhao K., Simpson D., Slater M.R., Urh M., Wood K.V. (2009). HaloTag7: A genetically engineered tag that enhances bacterial expression of soluble proteins and improves protein purification. Protein Exp. Purif..

[189] Oakley A.J., Prokop Z., Boháč M., Kmuníček J., Jedlička T., Monincová M., Kutá-Smatanová I., Nagata Y., Damborský J., Wilce M.C.J. (2002). Exploring the structure and activity of haloalkane dehalogenase from *Sphingomonas paucimobilis* UT26: Evidence for product- and water-mediated inhibition. Biochemistry.

[190] Xu D., Guo H. (2005). Electrostatic influence of active-site waters on the nucleophilic aromatic substitution catalyzed by 4-chlorobenzoyl-CoA dehalogenase. FEBS Lett..

[191] Jitsumori K., Omi R., Kurihara T., Kurata A., Mihara H., Miyahara I., Hirotsu K., Esaki N. (2009). X-Ray crystallographic and mutational studies of fluoroacetate dehalogenase from *Burkholderia* sp. strain FA1. J. Bacteriol..

[192] Satpathy R., Konkimalla V.B., Ratha J. (2015). In-silico Rational Protein Engineering and Design Approach to Improve Thermostability of a Haloalkane Dehalogenase Enzyme. Am. J. Bioinform..

[193] Noor S., Changey F., Oakeshott J.G., Scott C., Martin-Laurent F. (2014). Ongoing functional evolution of the bacterial atrazine chlorohydrolase AtzA. Biodegradation.

[194] Noor S., Taylor M.C., Russell R.J., Jermiin L.S., Jackson C.J., Oakeshott J.G., Scott C. (2012). Intramolecular Epistasis and the Evolution of a New Enzymatic Function. PLoS ONE.

[195] Banta S. Protein Engineering for Bioelectrocatalysis: We can do more than Vmax. Proceedings of the 217th ECS Meeting.

[196] Wang Y., Li X., Chen X., Chen D. (2013). Directed evolution and characterization of atrazine chlorohydrolase variants with enhanced activity. Biochemistry.

[197] Seffernick J.L., McTavish H., Osborne J.P., De Souza M.L., Sadowsky M.J., Wackett L.P. (2002). Atrazine chlorohydrolase from *Pseudomonas* sp. strain ADP is a metalloenzyme. Biochemistry.

[198] Lu Y., Yeung N., Sieracki N., Marshall N.M. (2009). Design of Functional Metalloproteins. Nature.

[199] Schanstra J.P., Janssen D.B. (1996). Kinetics of halide release of haloalkane dehalogenase: Evidence for a slow conformational change. Biochemistry.

[200] Abdul Hamid A.A., Tengku Abdul Hamid T.H., Abdul Wahab R., Omar M.S.S., Huyop F. (2015). An S188V Mutation Alters Substrate Specificity of Non-Stereospecific α-Haloalkanoic Acid Dehalogenase E (DehE). PLoS ONE.

[201] Hamid A.A.A., Wong E.L., Joyce-Tan K.H., Shamsir M.S., Hamid T.H.T.A., Huyop F. (2013). Molecular modelling and functional studies of the non-stereospecific α -haloalkanoic acid dehalogenase (DehE) from *Rhizobium* sp. RC1 and its association with 3-chloropropionic acid (β-Chlorinated Aliphatic acid). Biotechnol. Biotechnol. Equip..

[202] Hamid A.A.A., Hamid T.H.T.A., Wahab R.A., Huyop F. (2015). Identification of functional residues essential for dehalogenation by the non-stereospecific α-Haloalkanoic acid dehalogenase from *Rhizobium* sp. RC1. J. Basic Microbiol..

[203] Banáš P., Otyepka M., Jeřábek P., Petřek M., Damborsky J. (2006). Mechanism of enhanced conversion of 1,2,3-Trichloropropane by mutant Haloalkane dehalogenase revealed by molecular modeling. J. Comput. Aided Mol. Des..

[204] Bosma T., Damborsk J., Stucki G., Janssen D.B. (2002). Biodegradation of 1,2,3-Trichloropropane through Directed Evolution and Heterologous Expression of a Haloalkane Dehalogenase Gene. Appl. Environ. Microbiol..

[205] Pavlova M., Klvana M., Prokop Z., Chaloupkova R., Banas P., Otyepka M., Wade R.C., Tsuda M., Nagata Y., Damborsky J. (2009). Redesigning dehalogenase access tunnels as a strategy for degrading an anthropogenic substrate. Nat. Chem. Biol..

[206] Stepankova V., Damborsky J., Chaloupkova R. (2013). Organic co-solvents affect activity, stability and enantioselectivity of haloalkane dehalogenases. Biotechnol. J..

[207] Stepankova V., Khabiri M., Brezovsky J., Pavelka A., Sykora J., Amaro M., Minofar B., Prokop Z., Hof M., Ettrich R. (2013). Expansion of access tunnels and active-site cavities influence activity of haloalkane dehalogenases in organic cosolvents. Chembiochem.

[208] Manickam N., Mau M., Schlömann M. (2006). Characterization of the novel HCH-degrading strain, *Microbacterium* sp. ITRC1. Appl. Microbiol. Biotechnol..

[209] Magnuson J.K., Stern R.V., Gossett J.M., Zinder S.H., Burris D.R. (1998). Reductive dechlorination of tetrachloroethene to ethene by a two-component enzyme pathway. Appl. Environ. Microbiol..

[210] Maymó-Gatell X., Chien Y., Gossett J.M., Zinder S.H. (1997). Isolation of a bacterium that reductively dechlorinates tetrachloroethene to ethene. Science.

[211] Davis C.K., Webb R.I., Sly L.I., Denman S.E., Mcsweeney C.S. (2012). Isolation and survey of novel fluoroacetate-degrading bacteria belonging to the phylum Synergistetes. FEMS Microbiol. Ecol..

[212] Leigh J.A., Skinner A.J., Cooper R.A. (1988). Partial purification, stereospecificity and stoichiometry of three dehalogenases from a *Rhizobium* species. FEMS Microbiol. Lett..

[213] Slater J.H., Lovatt D., Weightman A.J., Senior E., Bull A.T. (1979). The growth of *Pseudomonas* putida on chlorinated aliphatic acids and its dehalogenase activity. J. Gen. Microbiol..

[214] Brokamp A., Schmidt F.R.J. (1991). Survival of *Alcaligenes xylosoxidans* degrading 2,2-dichloropropionate and horizontal transfer of its halidohydrolase gene in a soil microcosm. Curr. Microbiol..

[215] Senior E., Bull A.T., Slater J.H. (1976). Enzyme evolution in a microbial community growing on the herbicide Dalapon. Nature.

[216] Huang J., Xin Y., Zhang W. (2011). Isolation, characterization and identification of a *Paracoccus* sp. 2-haloacid-degrading bacterium from the marine sponge Hymeniacidon perlevis. J. Basic Microbiol..

[217] Kerr L.M., Marchesi J.R. (2006). Isolation of novel bacteria able to degrade α-halocarboxylic acids by enrichment from environmental samples. Chemosphere.

[218] He J., Ritalahti K.M., Aiello M.R., Löffler F.E. (2003). Complete detoxification of vinyl chloride by an anaerobic enrichment culture and identification of the reductively dechlorinating population as a Dehalococcoides species. Appl. Environ. Microbiol..

[219] Hendrickson E.R., Payne J.A., Young R.M., Starr M.G., Perry M.P., Fahnestock S., Ebersole R.C., Ellis D.E. (2002). Molecular Analysis of Dehalococcoides 16S Ribosomal DNA from throughout North America and Europe. Appl. Environ. Microbiol..

[220] Van Doesburg W., Van Eekert M.H.A., Middeldorp P.J.M., Balk M., Schraa G., Stams A.J.M. (2005). Reductive dechlorination of β-hexachlorocyclohexane (β-HCH) by a Dehalobacter species in coculture with a *Sedimentibacter* sp.. FEMS Microbiol. Ecol..

[221] Adrian L., Manz W., Szewzyk U., Görisch H. (1998). Physiological characterization of a bacterial consortium reductively dechlorinating 1,2,3- and 1,2,4-trichlorobenzene. Appl. Environ. Microbiol..

[222] Yu F., Cangelosi V.M., Zastrow M.L., Tegoni M., Plegaria J.S., Tebo A.G., Mocny C.S., Ruckthong L., Qayyum H., Pecoraro V.L. (2014). Protein Design: Toward Functional Metalloenzymes. Chem. Rev..

[223] Degtyarenko K. (2000). Bioinorganic motifs: Towards functional classification of metalloproteins. Bioinform. Rev..

[224] Finkelstein J. (2009). Metalloproteins. Nature.

[225] Waldron K.J., Rutherford J.C., Ford D., Robinson N.J. (2009). Metalloproteins and metal sensing. Nature.

[226] Holm R.H., Kennepohl P., Solomon E.I. (1996). Structural and Functional Aspects of Metal Sites in Biology. Chem. Rev..

[227] Palm-Espling M.E., Niemiec M.S., Wittung-Stafshede P. (2012). Role of metal in folding and stability of copper proteins in vitro. Biochim. Biophys. Acta.

[228] Song W.J., Sontz P.A., Ambroggio X.I., Tezcan F.A. (2014). Metals in Protein–Protein Interfaces. Annu. Rev. Biophys..

[229] Krishna S.S., Majumdar I., Grishin N.V. (2003). Structural classification of zinc fingers: Survey and summary. Nucleic Acids Res..

[230] Auld D.S. (2006). Structural Zinc Sites. Handbook of Metalloproteins.

[231] Strong L.C., McTavish H., Sadowsky M.J., Wackett L.P. (2000). Field-scale remediation of atrazine-contaminated soil using recombinant *Escherichia coli* expressing atrazine chlorohydrolase. Environ. Microbiol..

[232] Zastrow M.L., Peacock A.F.A., Stuckey J.A., Pecoraro V.L. (2012). Hydrolytic catalysis and structural stabilization in a designed metalloprotein. Nat. Chem..

[233] Berks B.C. (1996). A common export pathway for proteins binding complex redox cofactors?. Mol. Microbiol..

[234] Parmar A.S., Pike D., Nanda V. (2014). Computational Design of Metalloproteins. Methods in Molecular Biology.

[235] Hellinga H.W., Richards F.M. (1991). Construction of new ligand binding sites in proteins of known structure: I. Computer-aided modeling of sites with pre-defined geometry. J. Mol. Biol..

[236] Clarke N.D., Yuan S. (1995). Metal search: A computer program that helps design tetrahedral metal-binding sites. Proteins Struct. Funct. Genet..

[237] Stucki G., Thüer M. (1995). Experiences of a Large-Scale Application of 1,2-Dichloroethane Degrading Microorganisms for Groundwater Treatment. Environ. Sci. Technol..

[238] Alamo-Bethencourt V., Aldridge S., Coombs A., Defrancesco L., Huggett B., Osborne R. (2007). Mustard gas enzyme (News in brief). Nat. Biotechnol..

[239] Prokop Z., Opluštil F., DeFrank J., Damborský J. (2006). Enzymes fight chemical weapons. Biotechnol. J..

[240] Pieters R.J., Lutje Spelberg J.H., Kellogg R.M., Janssen D.B. (2001). The enantioselectivity of haloalkane dehalogenases. Tetrahedron Lett..

[241] Westerbeek A., Szymański W., Feringa B.L., Janssen D.B. (2011). Dynamic kinetic resolution process employing haloalkane dehalogenase. ACS Catal..

[242] Los G.V., Encell L.P., McDougall M.G., Hartzell D.D., Karassina N., Zimprich C., Wood M.G., Learish R., Ohana R.F., Urh M. (2008). HaloTag: A novel protein labeling technology for cell imaging and protein analysis. ACS Chem. Biol..

[243] Weimer E.P., Rao E., Brendel M. (1993). Molecular structure and genetic regulation of SFA, a gene responsible for resistance to formaldehyde in *Saccharomyces cerevisiae*, and characterization of its protein product. Mol. Gen. Genet..

[244] Yusn T.Y., Huyop F. (2009). Degradation of 3-Chloropropionic Acid by *Escherichia coli* JM109 Expressing Dehalogenase (deh) Gene used as Selection Marker. Biotechnology.

[245] Swanson P.E. (1999). Dehalogenases applied to industrial-scale biocatalysis. Curr. Opin. Biotechnol..

[246] Wiegel J., Wu Q. (2000). Microbial reductive dehalogenation of polychrorinated biphenyls. FEMS Microbiol. Ecol..

[247] Mena-Benitez G.L., Gandia-Herrero F., Graham S., Larson T.R., McQueen-Mason S.J., French C.E., Rylott E.L., Bruce N.C. (2008). Engineering a Catabolic Pathway in Plants for the Degradation of 1,2-Dichloroethane. Plant Physiol..

[248] LeBel G.L., Benoit F.M., Williams D.T. (1997). A one-year survey of halogenated disinfection by-products in the distribution system of treatment plants using three different disinfection processes. Chemosphere.

[249] Williams D.T., LeBel G.L., Benoit F.M. (1997). Disinfection by-products in Canadian drinking water. Chemosphere.

[250] Zhang P., Hozalski R.M., Leach L.H., Camper A.K., Goslan E.H., Parsons S.A., Xie Y.F., Lapara T.M. (2009). Isolation and characterization of haloacetic acid-degrading *Afipia* spp. from drinking water. FEMS Microbiol. Lett..

[251] Lauble H., Kennedy M.C., Emptage M.H., Beinert H., Stout C.D. (1996). The reaction of fluorocitrate with aconitase and the crystal structure of the enzyme-inhibitor complex. Proc. Natl. Acad. Sci. USA.

[252] Bunge M., Lechner U. (2009). Anaerobic reductive dehalogenation of polychlorinated dioxins. Appl. Microbiol. Biotechnol..

[253] Liu H., Park J.W., Häggblom M.M. (2014). Enriching for microbial reductive dechlorination of polychlorinated dibenzo-p-dioxins and dibenzofurans. Environ. Pollut..

[254] Fennell D.E., Nijenhuis I., Wilson S.F., Zinder S.H., Häggblom M.M. (2004). *Dehalococcoides ethenogenes* Strain 195 Reductively Dechlorinates Diverse Chlorinated Aromatic Pollutants. Environ. Sci. Technol..

[255] Liu F., Fennell D.E. (2008). Dechlorination and Detoxification of 1,2,3,4,7,8-Hexachlorodibenzofuran by a Mixed Culture Containing *Dehalococcoides ethenogenes* Strain 195. Environ. Sci. Technol..

[256] Rodenburg L.A., Du S., Lui H., Guo J., Oseagulu N., Fennell D.E. (2012). Evidence for dechlorination of polychlorinated biphenyls and polychlorinated dibenzo-p-dioxins and -furans in wastewater collection systems in the New York Metropolitan Area. Environ. Sci. Technol..

[257] Ahn Y.B., Häggblom M.M., Kerkhof L.J. (2007). Comparison of anaerobic microbial communities from Estuarine sediments amended with halogenated compounds to enhance dechlorination of 1,2,3,4-tetrachlorodibenzo-p-dioxin. FEMS Microbiol. Ecol..

[258] Vargas C., Fennell D., Häggblom M. (2001). Anaerobic reductive dechlorination of chlorinated dioxins in estuarine sediments. Appl. Microbiol. Biotechnol..

[259] Ahn Y.B., Liu F., Fennell D.E., Häggblom M.M. (2008). Biostimulation and bioaugmentation to enhance dechlorination of polychlorinated dibenzo-p-dioxins in contaminated sediments. FEMS Microbiol. Ecol..

[260] Bunge M., Adrian L., Kraus A., Opel M., Lorenz W.G., Andreesen J.R., Görisch H., Lechner U. (2003). Reductive dehalogenation of chlorinated dioxins by an anaerobic bacterium. Nature.

[261] Liu F., Cichocka D., Nijenhuis I., Richnow H.H., Fennell D.E. (2010). Carbon isotope fractionation during dechlorination of 1,2,3,4-tetrachlorodibenzo-p-dioxin by a Dehalococcoides-containing culture. Chemosphere.

[262] Bunge M., Wagner A., Fischer M., Andreesen J.R., Lechner U. (2008). Enrichment of a dioxin-dehalogenating *Dehalococcoides* species in two-liquid phase cultures. Environ. Microbiol..

[263] Mäntynen S., Rantalainen A.L., Häggblom M.M. (2017). Dechlorinating bacteria are abundant but anaerobic dechlorination of weathered polychlorinated dibenzo-*p*-dioxins and dibenzofurans in contaminated sediments is limited. Environ. Pollut..

[264] Hesseler M., Bogdanović X., Hidalgo A., Berenguer J., Palm G.J., Hinrichs W., Bornscheuer U.T. (2011). Cloning, functional expression, biochemical characterization, and structural analysis of a haloalkane dehalogenase from *Plesiocystis pacifica* SIR-1. Appl. Microbiol. Biotechnol..

[265] Fortova A., Sebestova E., Stepankova V., Koudelakova T., Palkova L., Damborsky J., Chaloupkova R. (2013). DspA from Strongylocentrotus purpuratus: The first biochemically characterized haloalkane dehalogenase of non-microbial origin. Biochimie.

[266] Ghaffar A., Tabata M. (2010). Enhanced dechlorination of chlorobenzene compounds on fly ash: Effects of metals, solvents, and temperature. Green Chem. Lett. Rev..

[267] Schmidberger J.W., Wilce J.A., Weightman A.J., Whisstock J.C., Wilce M.C.J. (2008). The Crystal Structure of DehI Reveals a New α-Haloacid Dehalogenase Fold and Active-Site Mechanism. J. Mol. Biol..

[268] Bedard D.L. (2004). Polychlorinated Biphenyls in Aquatic Sediments: Environmental Fate and Outlook for Biological Treatment. Dehalogenation.

[269] Pepino M., Tiemann C., Patterson B., Wice B., Klein S. (2013). Sucralose affects glycemic and hormonal responses to an oral glucose load. Diabetes Care.

[270] Dong S., Liu G., Hu J., Zheng M. (2013). Polychlorinated dibenzo-p-dioxins and dibenzofurans formed from sucralose at high temperatures. Sci. Rep..

[271] Wielgosiński G. (2010). The Possibilities of Reduction of Polychlorinated Dibenzo-P-Dioxins and Polychlorinated Dibenzofurans Emission. Int. J. Chem. Eng..

[272] Xiao Y., Jiang J., Huang H. (2014). Chemical dechlorination of hexachlorobenzene with polyethylene glycol and hydroxide: Dominant effect of temperature and ionic potential. Sci. Rep..

[273] Addink R., Olie K. (1995). Mechanisms of Formation and Destruction of Polychlorinated Dibenzo-*p*-dioxins and Dibenzofurans in Heterogeneous Systems. Environ. Sci. Technol..

[274] Gao Q., Cieplik M.K., Budarin V.L., Gronnow M., Jansson S. (2016). Mechanistic evaluation of polychlorinated dibenzo-p-dioxin, dibenzofuran and naphthalene isomer fingerprints in microwave pyrolysis of biomass. Chemosphere.

